# Multiscale modeling of bacterial colonies: how pili mediate the dynamics of single cells and cellular aggregates

**DOI:** 10.1088/1367-2630/aa5483

**Published:** 2017-01-10

**Authors:** Wolfram Pönisch, Christoph A Weber, Guido Juckeland, Nicolas Biais, Vasily Zaburdaev

**Affiliations:** 1 Max Planck Institute for the Physics of Complex Systems, D-01187 Dresden, Germany; 2 Paulson School of Engineering and Applied Sciences, Harvard University, Cambridge, MA 02138, USA; 3 Department of Information Services and Computing (FWC), Helmholtz-Zentrum Dresden-Rossendorf e.V, D-01314 Dresden, Germany; 4 Department of Biology, Brooklyn College, City University of New York, Brooklyn, NY 11210, USA; 5 Graduate Center of CUNY, NY 10016, USA

**Keywords:** bacterial mechanics, multicellular phenomena, biofilms, cell aggregation, *Neisseria gonorrhoeae*

## Abstract

*Neisseria gonorrhoeae* is the causative agent of one of the most common sexually transmitted diseases, gonorrhea. Over the past two decades there has been an alarming increase of reported gonorrhea cases where the bacteria were resistant to the most commonly used antibiotics thus prompting for alternative antimicrobial treatment strategies. The crucial step in this and many other bacterial infections is the formation of microcolonies, agglomerates consisting of up to several thousands of cells. The attachment and motility of cells on solid substrates as well as the cell–cell interactions are primarily mediated by type IV pili, long polymeric filaments protruding from the surface of cells. While the crucial role of pili in the assembly of microcolonies has been well recognized, the exact mechanisms of how they govern the formation and dynamics of microcolonies are still poorly understood. Here, we present a computational model of individual cells with explicit pili dynamics, force generation and pili–pili interactions. We employ the model to study a wide range of biological processes, such as the motility of individual cells on a surface, the heterogeneous cell motility within the large cell aggregates, and the merging dynamics and the self-assembly of microcolonies. The results of numerical simulations highlight the central role of pili generated forces in the formation of bacterial colonies and are in agreement with the available experimental observations. The model can quantify the behavior of multicellular bacterial colonies on biologically relevant temporal and spatial scales and can be easily adjusted to include the geometry and pili characteristics of various bacterial species. Ultimately, the combination of the microbiological experimental approach with the *in silico* model of bacterial colonies might provide new qualitative and quantitative insights on the development of bacterial infections and thus pave the way to new antimicrobial treatments.

## 1. Introduction

An essential step in the life of bacteria is the formation of microcolonies, agglomerates consisting of hundreds to thousands of cells. Microcolonies often are precursors to much more complex bacterial communities, known as biofilms [1, 2]. These early biofilms represent stable and tightly connected aggregates that can adhere to various substrates [3], such as epithelial cells [4] and medical catheters [5], or can grow on ship hulls [6] or inside of bioreactors [7]. In many cases, bacterial infections involving biofilms are much less responsive to antimicrobial treatments [8]. Thus, formation and control of biofilms is of high concern in medical and engineering applications. Many bacterial species rely on type IV pili, long polymeric semi-flexible filaments protruding out of the cell membranes, to attach to substrates [9, 10] and to interact with other bacterial cells. A few prominent examples of cells possessing pili and being involved in dangerous microbial infections are *Pseudomonas aeruginosa* [11], *Neisseria meningitidis* [12] and *Vibrio cholerae* [13]. A single pilus exhibits phases of elongation and retraction that produce pulling forces once a pilus is attached; a mechanism reminiscent of a grappling hook [14]. The forces are generated by the molecular motor PilT and are in the range of 100–180 pN corresponding to one of the strongest active molecular forces known in nature [15]. Pili mediated cell-to-substrate and cell-to-cell interactions were shown to be crucial for the formation and maintenance of microcolonies [16–20]. However, the exact mechanisms of how cells deploy pili to self-assemble into microcolonies and govern their internal dynamics are still poorly understood.

To scrutinize the role of the pili mediated cell-to-cell interactions driving microcolony formation we consider the example of the bacterium *Neisseria gonorrhoeae*. It is the causative agent of the second most common sexually transmitted disease, gonorrhea [21] and relies exclusively on pili appendages to move and agglomerate on surfaces. This makes it an ideal model system. Here, we present, to our knowledge, the first computational model of individual motile cells that move and interact via explicit pili dynamics and are able to agglomerate into stable microcolonies. Previous models either focused only on the dynamics of single cells due to individual pili [22, 23] or described the motility of colonies in a coarse-grained manner [19, 20]. Only recently individual-based modeling and experiments were combined in order to explain collective phenomena in microorganisms [24, 25]. Our model represents a versatile numerical tool that can be used to understand the behavior of *N. gonorrhoeae* on different length scales and allows us to make predictions about the dynamics of isolated cells and the dynamics of microcolonies. Highlights of the applications of our model include the detailed analysis of the dynamics of merging microcolonies and the understanding of the mechanisms responsible for the emergence of heterogeneous motility of cells within the colonies. Without tuning the model for a particular experimental condition, we obtain a qualitative agreement with experiment studies ranging from individual cells to colonies of several thousands of cells. For example, we can study the motility of colonies on a substrate or the differential motion of cells within a microcolony.

This paper is organized as follows. We present the computational model in section 2 and first use the model to describe the motility of an individual cell on a substrate (see section 3.1). We then turn to the motility of microcolonies on a substrate as a function of their size (see section 3.2). In section 3.3, we investigate the dynamics of individual cells within a microcolony and the structural properties of microcolonies. We show how the observed heterogeneity affects the dynamics of colony merging in section 3.4. In section 3.5, we study the self-assembly of multiple microcolonies on a surface and discuss the demixing of normal cells and a mutant having altered pili properties.

## 2. Computer model

Our model describes the interactions of individual cells by pili and is based on the current knowledge of the pili dynamics and their mechanism of force generation. Almost all parameters of the model can be determined experimentally (see below). In particular, in our model we describe the interactions of single cells via the binding of their individual pili to a substrate and to pili of other cells. By modeling the pili as dynamic springs that can protrude, retract, attach and detach, we can compute the forces acting on the cells and the resulting dynamics of single cells and microcolonies. The core properties of the model are discussed below while further details can be found in the supplementary information (see section S4).

### 2.1. Geometry of an individual cell and free pili dynamics

**Figure 1.**
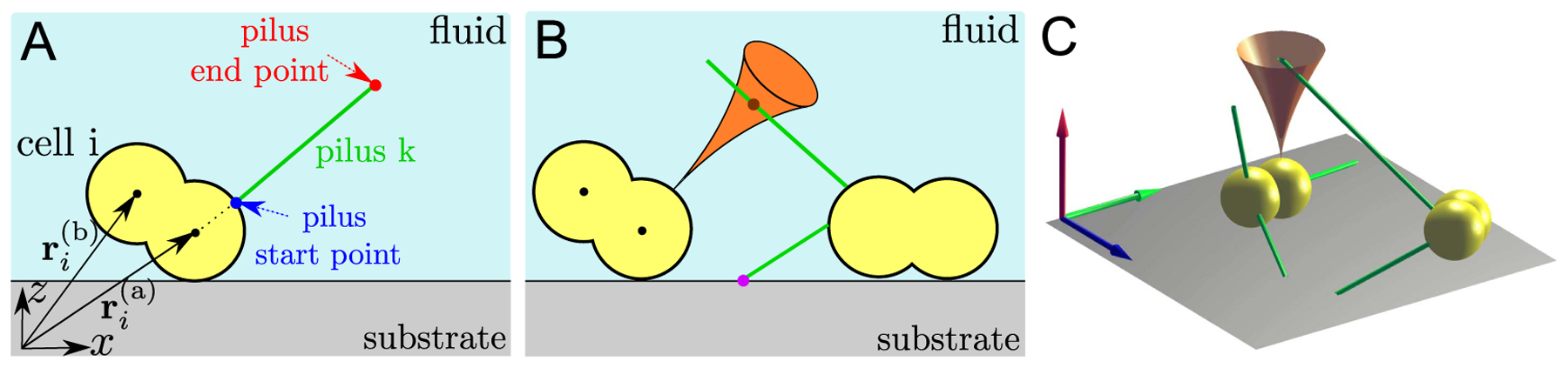
Schematic representation of pili dynamics and pili-mediated cell-substrate and cell-to-cell interactions. (A) Sketch of a diplococcus cell (yellow), modeled as two spherical cocci (a) and (b), on a substrate and surrounded by a fluid (blue). The centers of the two cocci are described by the position vectors $r_{i}^{\left(a\right)}$ and $r_{i}^{\left(b\right)}$. A pilus *k* (green) protrudes from the surface of the cell. The protrusion begins at the start point $x_{k}^{\left(\text{s}\right)}$ (blue dot) and the end point of the growing pilus at a certain moment in time is located at the position $x_{k}^{\left(\text{e}\right)}$ (red dot). (B) Illustration of pili-mediated interactions. Pili can bind to the substrate at a specific point (purple dot). Additionally, two pili can bind to each other. The binding probability is governed by the intersection of one pilus with a region (orange) obtained from solving the beam equation for the other pilus. The binding position (brown dot) of both pili is chosen randomly on the intersection line of the pilus and beam region. (C) Representation of the three-dimensional shape of the cells sitting on a substrate. The green lines represent the pili and the cone shows the solution of the beam-equation for a pilus (as discussed in (B)). The three arrows highlight the cartesian coordinate system, where the red arrow represents the *z*-direction.

The bacterium *Neisseria gonorrhoeae* has the shape of two overlapping spheres, referred to as a diplococcus (see figures 1(A) and (C)). We reconstitute the shape of cell *i in silico* by two spheres, called cocci (a) and (b), each with a radius *R* and positions of their centers $r_{i}^{\left(\text{a}\right)}$ and $r_{i}^{\left(\text{b}\right)}$. The two spheres are fixed at a distance $d=\left|r_{i}^{\left(a\right)}-r_{i}^{\left(b\right)}\right|$ [22]. The center of mass (COM) of cell *i* is defined as $r_{i}^{\left(\text{com}\right)}=\left(r_{i}^{\left(\text{a}\right)}+r_{i}^{\left(\text{b}\right)}\right)/2$.

A pilus is modeled as a spring, which is characterized by two points, its start point on the surface of a cell and an end point (see figure 1(A)). The contour length of the pilus is the distance between these two points. The characteristic length of a pilus is *l*_ch_ = 1.2 *μ*m [22] and it is considerably smaller than the persistence length *l*_p_ = 5.0 *μ*m [26], making it semiflexible.

An average cell possesses around 10–20 pili [22, 27], which are continuously assembled and disassembled. There is evidence that the number of pili is not only limited by the available number of monomers inside of the bacterial membrane, but instead the number of domains responsible for pili and their cycles of protrusion and retraction [28]. Thus, there is a maximal number of pili *N*_p_. In our model, we describe the dynamics of pili protrusion and retraction as a stochastic process. Pili begin to assemble randomly with a rate *λ*_p_ until a cell has a maximal number of pili, *N*_p_. The start point of the pilus *k* is randomly distributed on the surface of the diplococcus. Each pilus *k* protrudes perpendicularly from the surface of the diplococcus cell with a velocity *v*_pro_. The pilus switches from a protrusion state to a retraction state with a probability that corresponds to a rate *λ*_ret_. The speed of retraction of each free pilus *v*_ret_ is constant. If the end point of a perpendicularly protruding pilus is inside of the substrate, it will slide along the substrate. Additionally, there is no volume exclusion of pili and cells in our model. If the contour length of a free pilus has shrunk to zero, the pilus is removed.

### 2.2. Attachment to substrate or other pili

Pili can attach to the substrate or bind to pili of other cells. In our model binding to the substrate can occur after the pilus has touched the surface of the substrate. The binding dynamics is described by a stochastic process characterized by the rate *λ*_sub_. Once a pilus binds to the substrate it will attach with its end point to the surface of the substrate.

Pili are able to attach to pili of other cells, but not to the surface of the cells, as has been shown experimentally for *N. gonorrhoeae* [29]. To identify the contact point between two pili, we assume that one pilus swipes through a certain region in space due to thermal fluctuations (orange region in figure 1(B)). An approximation to this region is given by the solution to the beam-equation of a semiflexible rod [30]. Once another pilus overlaps with the region of the beam both can bind with a rate *λ*_pil_. The position of the binding point of the pili is randomly chosen on the intersection line of the pilus and the beam region (see figures 1(B) and (C)). Subsequently to a binding event of two pili or between a pilus and the substrate, each pilus begins to retract. While there is no direct evidence that pili binding triggers the retraction, it was suggested before [28, 31] and we make this assumption in the model.

### 2.3. Pili forces

Pili elongate and retract due to the assembly and disassembly of its subunits within the membrane of the cells [28]. The molecular motor PilT, involved in the disassembly, is able to produce pulling forces up to 100–180 pN [32]. In order to include this behavior in our model, pili are modeled as Hookean springs with a spring constant *k*_pull_ [33].

To compute the corresponding forces of actively pulling pili, we introduce a second length next to the contour length, the so called free length. It corresponds to the length of a pilus if it were not attached to the substrate or another pilus and thus the spring would not be under tension. While the free and the contour length of a pilus are equivalent for a non-attached pilus, in case of attachment to the substrate or another pilus, they do not need to be equal. While the contour length solely depends on the motion of the cells and the position of the pilus start and end points, the free length of the pilus is changed due to its retraction. From the difference between the contour and the free length one can compute the pulling force of a pilus (see supplementary information section S2). Similar to many other molecular motors, i.e. kinesin [34] or RNA polymerase [35], the PilT motor exhibits stalling. This means that the retraction velocity of a pilus *k* depends on its pulling force [32]:

(1) $$v_{k}^{\left(\text{ret}\right)}(F)=\text{max}\left[0,v_{\text{ret}}\left(1-\frac{F}{F_{\text{stall}}}\right)\right].$$

Here, *F* is either the absolute value of the pili–pili-forces $F=\left|{\text{F}}_{k}^{\left(\text{pp}\right)}\right|$, or the absolute value of a force resulting from an attachment to the substrate $F=\left|F_{k}^{\left(\text{ps}\right)}\right|$. *F*_stall_ is the stalling force and it determines the characteristic pulling force of a pilus [32]. Although each pilus motor operates independently, pili can simultaneously engage in pulling, thus providing a cooperative and additive effect, similar to what was previously reported for pili bundles [36].

The pulling force also affects the detachment probability of the pilus (forced unbinding). For the pili-substrate and pili–pili-bonds the detachment rates *λ*_d,sub_ and *λ*_d,pil_ are given by

(2) $$\lambda_{\text{d},\text{sub}}(F)=\frac{1}{\tau_{\text{d},\text{sub}}}\text{exp}\left(\frac{F}{F_{\text{d},\text{sub}}}\right),$$

(3) $$\lambda_{\text{d},\text{pil}}(F)=\frac{1}{\tau_{\text{d},\text{pil}}}\text{exp}\left(\frac{F}{F_{\text{d},\text{pil}}}\right).$$

Here, *F*_d,sub_ and *F*_d,pil_ are the corresponding characteristic detachment forces, *τ*_d,sub_ and *τ*_d,pil_ are the characteristic detachment times. The probability *P*_det_ for a pilus to detach during a small time interval Δ*t* is then *P*_det_ = *λ*_det_ Δ*t*. After detachment, the free pilus is able to rebind again to the substrate or other non-attached pili.

### 2.4. Cell forces and motility

**Figure 2.**
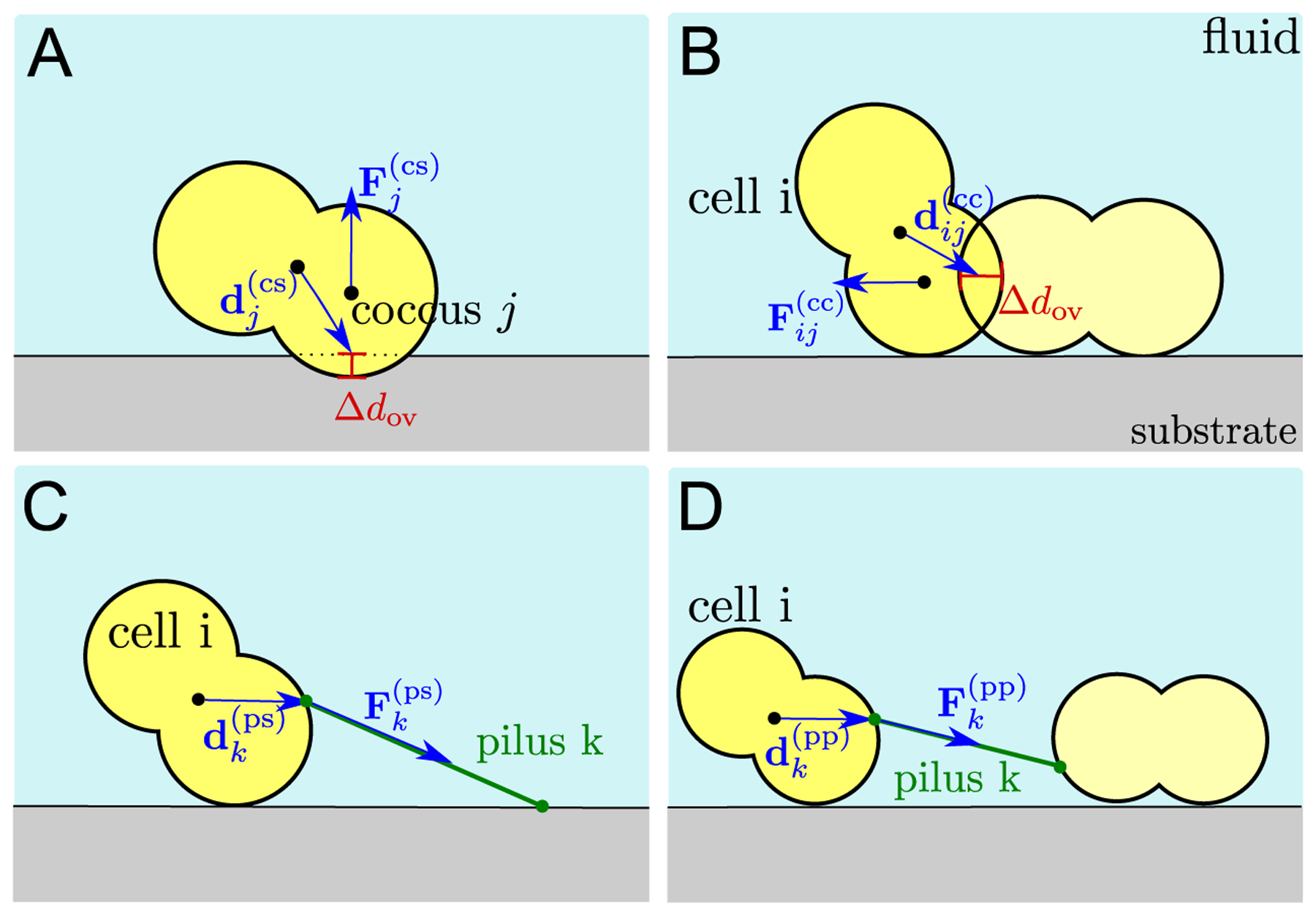
Sketch of forces acting on the cell. We distinguish between two different classes of forces, excluded volume forces (A) and (B) and pili-mediated forces (C) and (D). Excluded volume forces result from an overlap Δ*d*_ov_ of a coccus and a substrate (A) and an overlap of two cocci (B). Both effects mediate a repulsive force, $F_{j}^{\left(\text{cs}\right)}$ and $F_{ij}^{\left(\text{cc}\right)}$, that acts on the cell at the intersecting point given by the vectors $d_{j}^{\left(\text{cs}\right)}$ and $d_{ij}^{\left(\text{cc}\right)}$. Similarly, pili mediate forces due to pili-substrate bonds (C) or pili–pili bonds (D), $F_{k}^{\left(\text{ps}\right)}$ and $F_{k}^{\left(\text{pp}\right)}$, acting at the start points of the pili, characterized by the vectors $d_{k}^{\left(\text{ps}\right)}$ and $d_{k}^{\left(\text{pp}\right)}$.

The interplay of pili-mediated and excluded volume forces leads to translation and rotation of the cells. The forces acting on the cells are visualized in figure 2. An intersection of two cocci of two different cells causes a repulsive force, which we describe as a simple harmonic force. The total force acting on the COM $r_{i}^{\left(\text{com}\right)}$ of cell *i* results from multiple contributions: excluded volume forces $F_{j}^{\left(\text{cs}\right)}$ due to the intersection of the coccus *j* (of cell *i*) and the substrate (see figure 2(A)), excluded volume forces $F_{ij}^{\left(\text{cc}\right)}$ due to cells *j* overlapping with cell *i* (see figure 2(B)), forces of all pili *k* emerging from the cell and being attached to the substrate $F_{k}^{\left(\text{ps}\right)}$ (see figure 2(C)) and forces of all pili *k* emerging from the cell attached to other pili $F_{k}^{\left(\text{pp}\right)}$ (see figure 2(D)):

(4) $${\text{F}}_{i}^{\left(\text{tot}\right)}=\sum_{\text{j}}{\text{F}}_{ij}^{\left(\text{cc}\right)}+\sum_{\text{j}}{\text{F}}_{j}^{\left(\text{cs}\right)}+\sum_{\text{k}}{\text{F}}_{k}^{\left(\text{ps}\right)}+\sum_{\text{k}}{\text{F}}_{k}^{\left(\text{pp}\right)}.$$

Here, we only sum over the cocci and the pili of cell *i*. The velocity of cell *i* is related to the force by a friction coefficient *μ*_trans_ in the overdamped limit [37]:

(5) $$\frac{\text{d}r_{i}^{\left(\text{com}\right)}}{\text{d}t}=\frac{\text{d}r_{i}^{\left(\text{a}\right)}}{\text{d}t}=\frac{\text{d}r_{i}^{\left(\text{b}\right)}}{\text{d}t}=\mu_{\text{trans}}F_{i}^{\left(\text{tot}\right)}.$$

The rotation of the cell *i* is described in a similar manner. The total torque is given by

(6) $${\text{T}}_{i}^{\left(\text{tot}\right)}=\sum_{j}d_{ij}^{\left(\text{cc}\right)}\times {\text{F}}_{ij}^{\left(\text{cc}\right)}+\sum_{j}{\text{d}}_{j}^{\left(\text{cs}\right)}\times {\text{F}}_{j}^{\left(\text{cs}\right)}+\sum_{k}d_{k}^{\left(\text{ps}\right)}\times F_{k}^{\left(\text{ps}\right)}+\sum_{k}d_{k}^{\left(\text{pp}\right)}\times F_{k}^{\left(\text{pp}\right)}.$$

Again, we only sum over the cocci and the pili of cell *i*. The vectors $d_{ij}^{\left(\text{cc}\right)}$, $d_{j}^{\left(\text{cs}\right)}$, $d_{k}^{\left(\text{ps}\right)}$ and $d_{k}^{\left(\text{pp}\right)}$ are visualized in figure 2 and represent the vectors from the COM of the cell towards the point at which the forces act. The total torque allows to compute the angular velocity

(7) $$\omega_{i}=\mu_{\text{rot}}{\text{T}}_{i}^{\left(\text{tot}\right)},$$

which describes how the cocci positions and the pili start and end points rotate around the COM $r_{i}^{\left(\text{com}\right)}$ of the cell. Here *μ*_rot_ is the rotational mobility.

### 2.5. Simulation details and parameters

Our simulations were performed on the local computing cluster consisting of x86–64 GNU/Linux systems of the Max Planck Institute for the Physics of Complex Systems. The code was written in C++ and parallelized on CPU by using the library OpenMP. More details are given in section S2 in the supplementary information. In the simulations, the Euler algorithm with the time step Δ*t* = 5 × 10^−6^ s is used to solve the equations of motion (see equations (5) and (7)). We have checked that higher order schemes produce comparable results but do not provide any noticeable speedup.

Our model in total contains 19 parameters. 13 of those parameters (see table 1) are either known from literature or affect the outcome only weakly, i.e. the excluded volume constants *k*_cc_ and *k*_cs_ (as long as they are large enough to reduce overlapping of the cells) [38]. Below, we show that the excluded volume constant *k*_cc_ does not affect the results when varied in broad range. For the pilus production rate *λ*_p_ we picked a value that is in the order of previously published values [23]. The translational and rotational mobilities were chosen to be of the order of the mobility of a sphere with a diameter of 1 *μ*m moving in a liquid which is roughly 10 times more viscous than water. Increasing the viscosity of the surrounding medium allows us to increase the simulation time step and thus speed up the simulation. We expect no alteration of the simulation results because for the used mobility *μ*_trans_ a small net force of 2 pN is sufficient to create a motion of the cell with a velocity comparable to the retraction velocity *v*_ret_ of a pilus. For a viscosity similar to that of water, a force of only 0.2 pN is required. Both force values are much smaller than the characteristic forces of individual pili of the order of 100 pN. Additionally, for both viscosities only a few pili are required to produce pulling forces that correspond to the motion of large colonies consisting of up to thousands of cells with a velocity similar to the retraction velocity *v*_ret_. The remaining parameters were sampled to explore the behavior of the model (see table 2 and section S4 in the supplementary information).

For this paper we picked two different parameter sets characterizing the strength of the interactions between pili (see table 3). We refer to them as strong and weak characterized by different pili–pili-detachment forces *F*_d,pil._ The strong parameter set has a larger detachment force (360 pN), thus a larger force needs to act on a pilus in order to allow the bond to detach. For the weak parameter set, the corresponding detachment force is smaller (120 pN). While the strong parameter set is compatible with the experimental data on *N. gonorrhoeae*, weaker interactions may enable us to describe the behavior of other bacteria, for example *N. meningitidis*.

We have also chosen to represent the range of substrate interactions by two parameter sets, a strong and a weak one (see table 4). This is motivated by the use of different substrates (i.e. glass or plastic) and corresponds to different values of the pili-substrate detachment force *F*_d,sub_ ranging from 10 to 180 pN [27, 33, 39]. Different substrates may alter the motility of individual cells and microcolonies. Detailed information about the data analysis of the results are given in the supplementary information in section S5.

Although the pili–pili detachment forces *F*_d,pil_ in the order of 20–80 pN have been measured [27, 29], we used parameters (see table 3) that are larger roughly by a factor of 4. This choice was required for our model to be consistent with the existing experimental data on microcolony behavior (see section 3.4). There are several possible reasons for slightly higher detachment forces in our model. The exact detachment force between pili will depend on the geometric configuration of the two interacting pili. The ability of pili to form multiple attachments, i.e. in the form of bundles over some length [23, 36], increases the stability of the resulting cell to cell binding. Thus within the binary pili interaction approach an increased pili stability might require a higher detachment force. Additionally, *N. gonorrhoeae* can undergo pilin antigenic variations which affect the binding properties of pili and thus can alter the strength of pili–pili- and pili-substrate bonds. Thus, the exact pilin sequence needs to be controlled in experiments [22, 40]. Finally, the reported pili stalling forces vary in the range of 100–180 pN.We have chosen the maximal value of the stalling force *F*_stall_. For a lower stalling force of 100 pN, a correspondingly smaller detachment force of 50 pN would produce similar results to our weak parameter set, characterizing the pili–pili interactions. For completeness, we will also provide simulation results for the combination *F*_d,pil_ = 50 pN and *F*_stall_ = 180 pN (see below).

## 3. Results

The proposed model allows us to investigate the dynamics of *N. gonorrhoeae* and other bacteria using type IV pili on different scales, from individual cells to multiple colonies.

### 3.1. Surface-motility of a single cell

Previously, we have studied the pili-mediated motility of individual bacteria on a substrate [22]. For example, we observed a bimodality of the velocities and a pronounced correlation between the direction of motion and the orientation of the cell body [22]. Additionally, cells seem to exhibit a persistent motion on length scales larger than the average pili length [23, 27], thus suggesting cooperative interactions of multiple pili.

**Figure 3.**
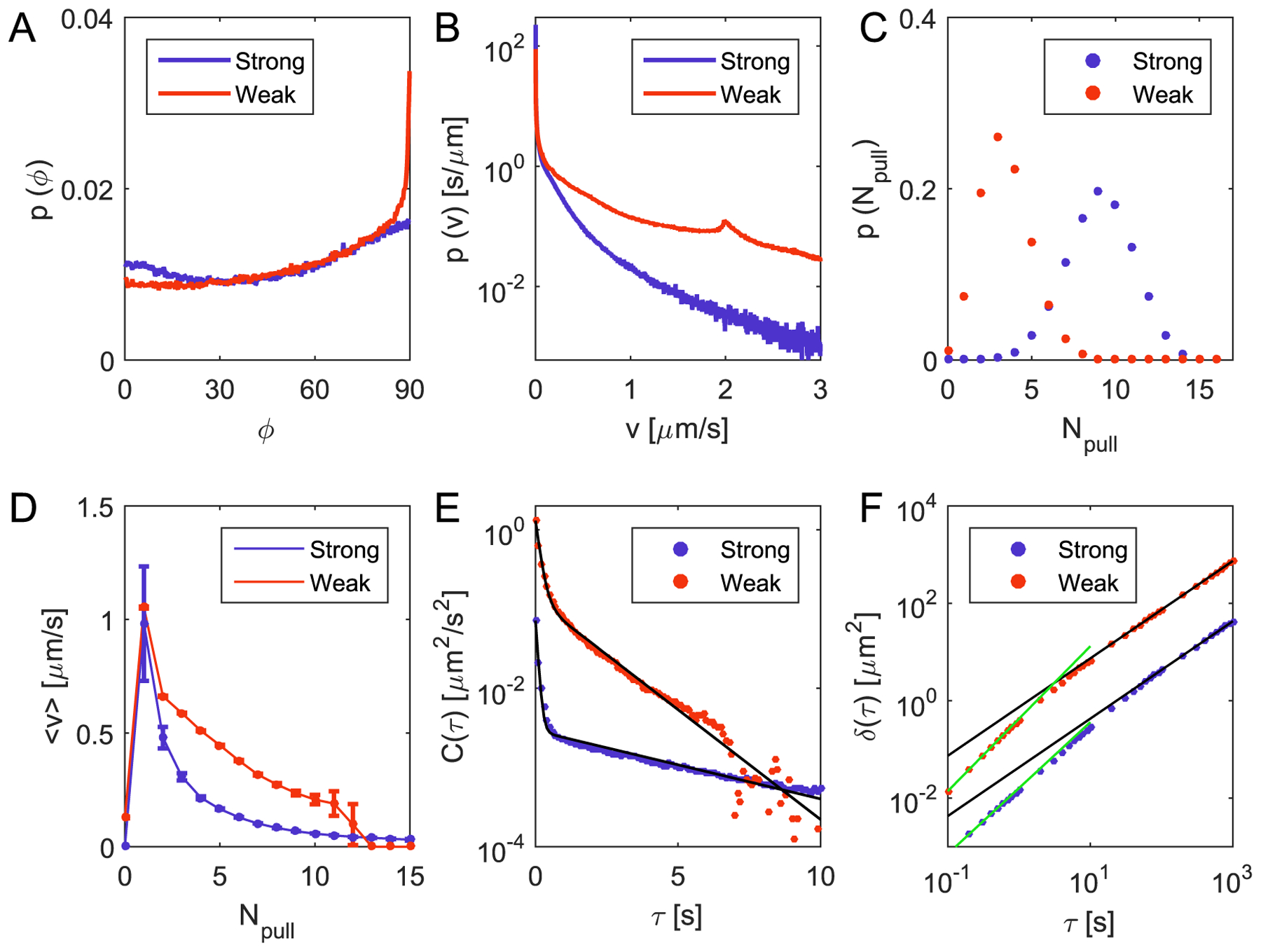
Motility of single cells moving over a substrate. Red and blue colors denote weak and strong parameter sets, respectively (see table 4). (A) Probability density function of the the angles between the velocity direction of the cell and its orientation (the axis between its cocci). For both parameter sets there is a preference towards 90°. (B) Probability density function of absolute velocities of the cell motion. For the weak surface interactions a small peak around 2 *μ*m s^−1^ is observed. (C) Probability density function of the number of pili producing a non-zero force due to attachment to the substrate. For strong attachments, the distribution is shifted to higher numbers. (D) Average absolute velocities as a function of the number of actively pulling pili. Although the two parameter sets highly affect the dynamics of individual cells on a substrate, both functions show a similar behavior. While the velocity is maximal for a single pilus, for higher numbers of pili, it decreases. (E) Velocity autocorrelation function as a function of time. The black lines represent double exponential fits (see section S3.1 in the supplementary information) with characteristic times of *τ*_1_ = (0.08 ± 0.01) s and *τ*_2_ = (5.05 ± 0.40) s for strong interactions and *τ*_1_ = (0.15 ± 0.04) s and *τ*_2_ = (1.56 ± 0.08) s for weak interactions. (F) Mean squared displacement as a function of time. The black lines represent a linear time dependence, thus describing asymptotic diffusive behavior. For times *τ* < 10 s, we observe a super-diffusive behavior with exponents (1.35 ± 0.11) for strong interactions and (1.49 ± 0.05) for weak interactions (see green lines).

In agreement with previous work [22], in our model we also observe a bias towards 90° for the angle between the cell orientation and direction of motion for a wide range of parameters (see figure 3(A)). This effect was attributed to the geometry of the cell: more pili are involved in pulling on the longer side of the cell. Additionally, we can study the histogram of cell speeds (see figure 3(B)) for which a bimodal behavior has been shown previously. The velocities result from the displacement of cells in the plane tangential to the substrate (see section S5.1 in the supplementary information). We observe a peak at zero velocity, corresponding to either a lack of pili attached to the substrate or a high number of attached pili pulling against each other in a situation similar to a tug-of-war [23]. For weak substrate interactions, we detect a peak around 2 *μ*m s^−1^ in the distribution of velocities, corresponding to the pulling of a single pilus, for which we set *v*_ret_ = 2 *μ*m s^−1^. No such peak can be seen for stronger interactions (see figure 3(B)). For this parameter set (strong), we observe a reduction of the average velocity. By computing the average number of actively pulling pili, we observe an increase in the number of pili participating in the tug-of-war (see figure 3(C)). We suggest that this increase is the reason for the absence of a peak around 2 *μ*m s^−1^. The probability of the attachment of only a single pilus to the substrate is lower compared to the case of weaker interactions. Thus, it is more likely that a higher number of pili is actively pulling, which corresponds to smaller mean velocities (see figure 3(D)). The dependence of the velocity and the pili number is robust against the use of different parameter sets (see figure 3(D)) and it decreases for an increasing number of pili.

Moreover, we observe a non-trivial behavior of the velocity autocorrelation function *C* (*τ*), which can be well described by a double-exponential form (see figure 3(E)):

(8) $$C(\tau )={\langle v(t+\tau )\cdot v(t)\rangle }_{t}=v_{1}^{2}\cdot \text{exp}\left(-\frac{\tau }{\tau_{1}}\right)+v_{2}^{2}\cdot \text{exp}\left(-\frac{\tau }{\tau_{2}}\right).$$

The short correlation times *τ*_1_ of the order of 0.1 s reflect rapid reorientations of cells due to newly attaching and detaching pili, while the longest times *τ*_2_ reflect a persistence of motion on the time scale of several pili cycles (see table S1 in the supplementary information). The larger time *τ*_2_ captures the crossover time between the super-diffusive to diffusive scaling of the mean squared displacement (see figure 3(F)).

Altogether, our results show that the number of bound pili, which is influenced by the properties of the substrate, determines the cell motion on the substrate. Increasing the number of pili or the strength of their attachment decreases the average speed of cells. Stronger attachment also leads to longer periods of persistent motion.

### 3.2. Surface-motility of a microcolony

**Figure 4.**
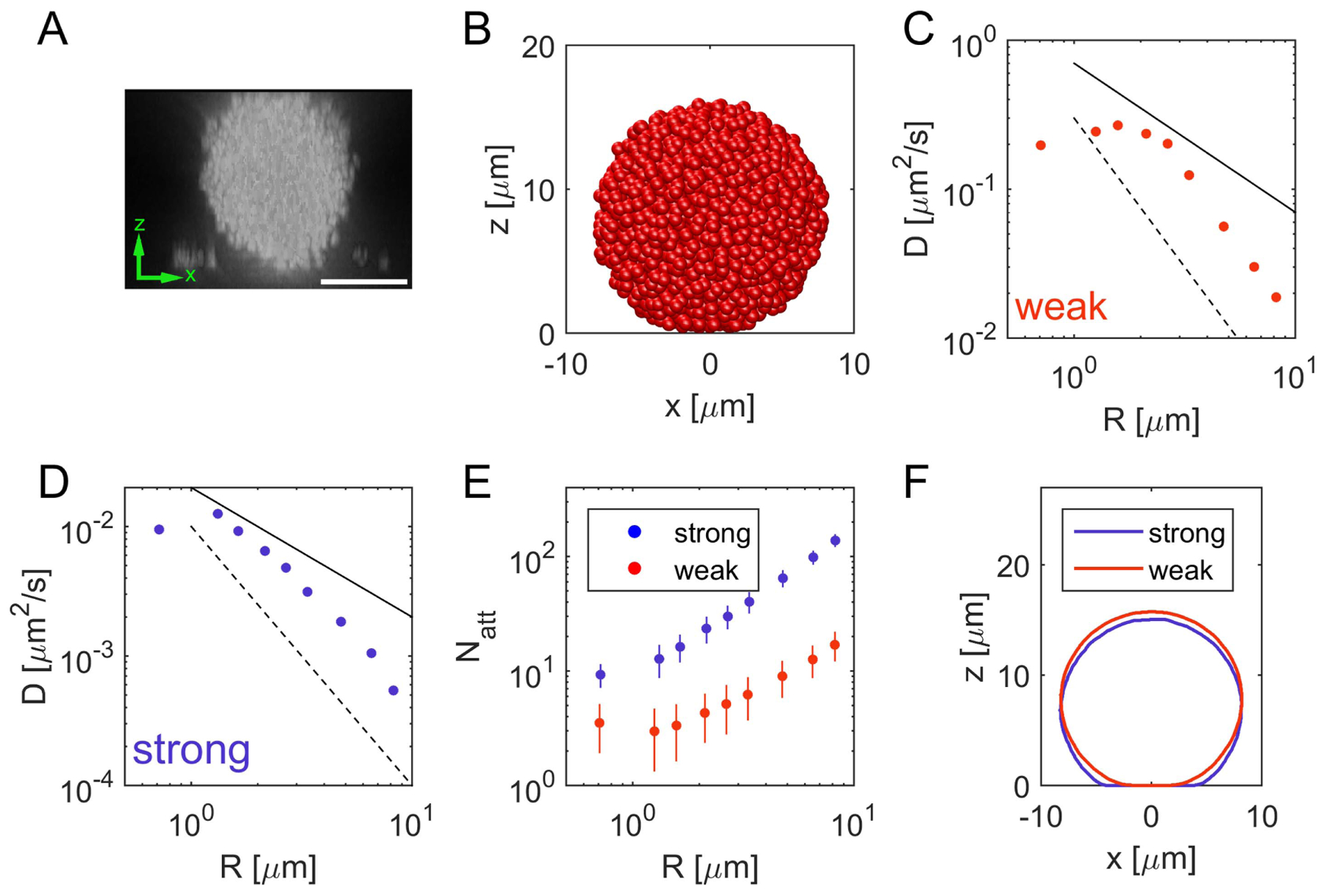
Properties of microcolonies on a surface. We only consider strong pili–pili-interactions (see table 3) and analyze the properties for strong and weak substrate interactions (see table 4). (A) Microscopic image of the three-dimensional shape of a fixed *N. gonorrhoeae* microcolony on a substrate from confocal microscope images (scale bar = 10 *μ*m). (B) Image of a *in silico* microcolony on a surface, defined by *z* = 0 for weak pili-substrate interactions. (C), (D) Diffusion coefficient as a function of the colony radius for weak (C) and strong (D) substrate interactions. The solid black line represents a power law with exponent −1 (Stokes-Einstein-relation [41]), whereas the dashed line corresponds to a power law with the exponent −2. Combined with our result that a reduced number of pili increases the motility of single cells, the initial increase of the diffusion coefficient rises due to the binary nature of pili–pili interactions in the model. Pili–pili-bundles reduce the number of available pili-substrate interactions. (E) Number of pili attached to the substrate as a function of the colony radius. The larger the colony, the more pili are attached to the substrate. (F) Shape of an individual colony consisting of 1600 cells on a surface (please refer to supplementary information section S5.2 for details). For stronger pili-substrate interactions the colony increases its contact area to the surface.

During the infection process of *N. gonorrhoeae*, the interplay between the forming microcolonies and the substrate (see figure 4(A)) plays an essential role [4]. One of the major mechanisms of how colonies grow is the coalescence of two smaller colonies [19] (see section 3.4 in the supplementary information). However, the precondition for coalescence is that colonies are close to each other. Therefore, they use pili to move over the substrate and find their merging partners just like individual cells.

To study the motility of microcolonies over a substrate, we initiated individual spherical colonies attached to a substrate for different values of the detachment forces and times of pili-substrate bonds (see figure 4(B) and section S4.2 in the supplementary information). By measuring the mean squared displacement of differently sized colonies as a function of time, we calculated their diffusion constants as a function of the colony size (see section S5.2 in the supplementary information). In agreement with experiments [19], we observe a scaling *D* ∝ *R*^−*α*^ with *α* > 1(see figures 4(C), (D) and figure S1 in supplementary information). Thus, larger colonies exhibit a decreased motility. Furthermore, we observe that the diffusion coefficients have the same order of magnitude as the values previously measured for the motion of colonies on a glass surface [19]. Moreover, we observe a correlation between the decrease of the diffusion coefficient and an increase of the number of attached pili (see figure 4(E)). This behavior originates from the same mechanisms as the decreasing velocity of individual cells with an increasing number of pulling pili (see section 3.1). The more pili participate in the tug-of-war, the more the colony is trapped and the weaker is its motility.

Besides the motility of microcolonies moving over a substrate, our model also enables us to study the ‘wetting’ behavior of microcolonies [42]. Experiments show that *N. gonorrhoeae* colonies on a substrate maintain an almost spherical shape (see figure 4(A)). The shape of the colony results from a competition between the interactions of cells within the colony and the interactions of the colony with the substrate. This is confirmed in our simulations, where we observe an almost spherical shape of colonies for strong pili–pili-interactions (see figure 4(F) and section S5.2 in the supplementary information). This shape is altered for an increasing interaction strength with the substrate: in this case the colony increases its contact area with the surface, which is reminiscent of partial wetting.

To summarize, we observe a decreasing diffusion coefficient of microcolonies on a surface as a function of their size, in agreement with previous experimental measurements [19]. Additionally, we can employ our model to study the shape and the wetting behavior of microcolonies as a function of their substrate interactions. In the future, we can also study the effects of external forces or flows.

### 3.3. Internal dynamics of individual colonies

**Figure 5.**
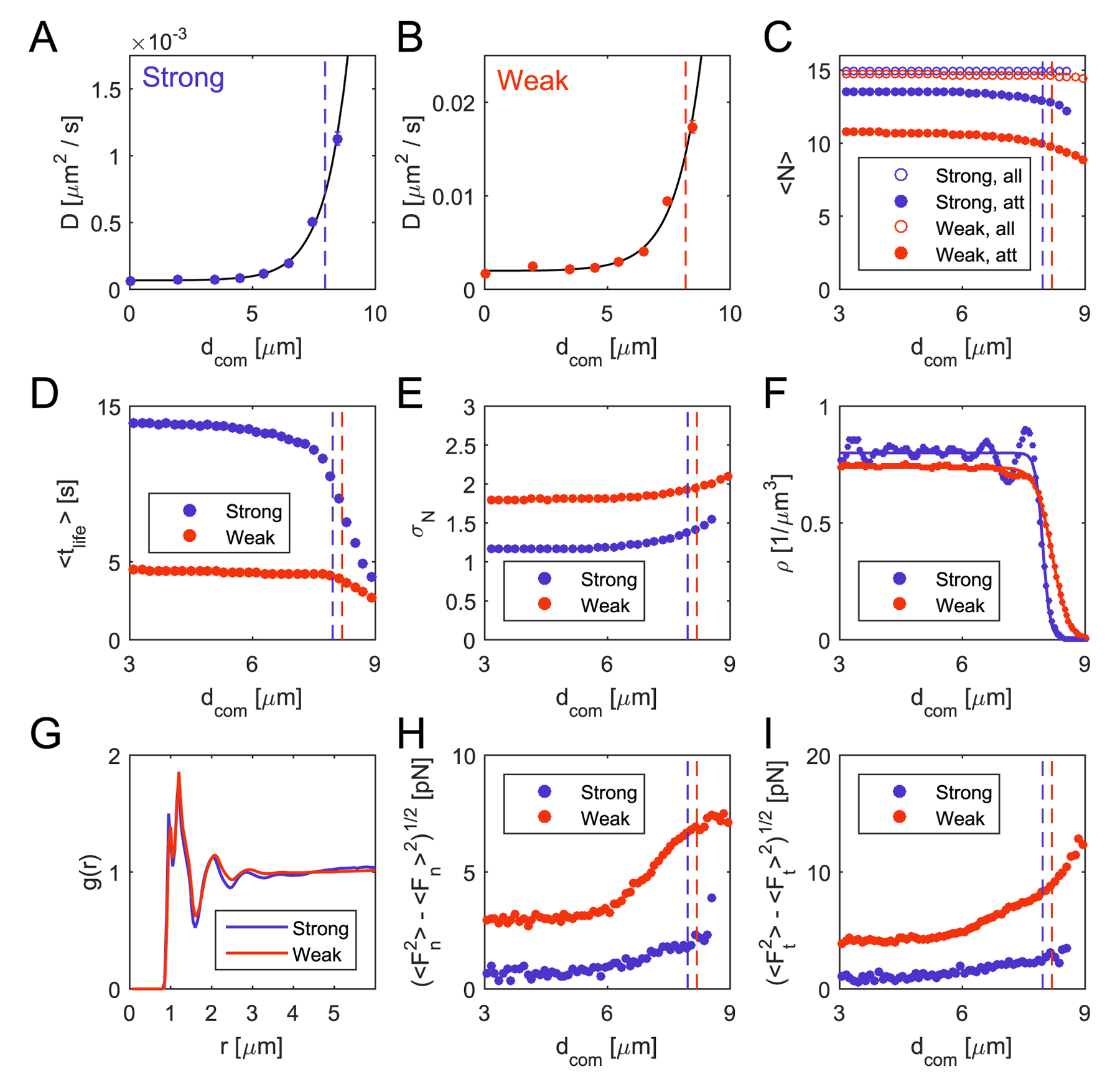
Internal properties of individual colonies for strong and weak pili–pili-interactions in the absence of a substrate (see table 3). Vertical dashed lines represent the corresponding radii of the colonies *R*_col_ by fitting the density profile of cells within the microcolony according to equations (10). (A), (B) Diffusion coefficient as a function of the distance *d*_com_ from the center of the colony for an individual cell. We observe a strong gradient for both parameter sets, which can be characterized by equation (9). For the strong and weak parameter sets we compute characteristic length scales of (0.98 ± 0.17) *μ*m (A) and (1.06 ± 0.31) *μ*m, respectively (B). (C) Mean of the number of all pili, and of the pili which generate a pulling force on cells as a function of *d*_com_. (D) Mean life time of the pili of cells within a microcolony. For strong pili–pili interactions individual pili exists for a considerably longer time. (E) Standard deviation of the number of all pili, and of the pili which generate a pulling force on cells as a function of *d*_com_. (F) Cell number density *ρ* of cells as a function of the distance *d*_com_ from the center of the colony. The lines represent fits with a tanh-function, which is also a solution of the interfacial profiles in phase separated binary mixtures [43]. (G) Pair-correlation function of the centers of cells within a colony. The first peak consists of two individual peaks resulting from the diplococcus shape of the cells. (H) Standard deviation of the normal net forces acting on a cell relative to the surface of the colony. The fluctuations are more pronounced close to the surface of the colony. (I) Standard deviation of the tangential net forces acting on a cell relative to the surface of the colony. The fluctuations are stronger close to the surface of the colony.

By considering ‘wetting’ of colonies on the substrate, we implicitly assumed that the microcolonies were liquid-like. To scrutinize this assumption, we investigate the behavior of individual cells within a colony. By measuring the time-averaged mean squared displacement $\delta (\tau )={\langle {\left[r_{i}^{\left(\text{com}\right)}(t+\tau )-r_{i}^{(\text{com) }}(t)\right]}^{2}\rangle }_{t}$ of the trajectories of individual cells in our model, we can confirm the experimental observation that cells at the colony boundary are significantly more motile (see figures 5(A), (B) and S2, section S5.3 in the supplementary information). To determine the characteristic length scale *d*_grad_ of the gradient of the diffusion coefficient, we can use an exponential fit to the dependence of *D* on the distance from the colony center *d*_com_,

(9) $$D\left(d_{\text{com}}\right)=D_{0}+D_{r}\text{exp}\left(\frac{d_{\text{com}}}{d_{\text{grad}}}\right).$$

Here, *D*_0_ corresponds to a random motion of the cells independent of the position within a colony, *D*_r_ characterizes the magnitude of the gradient of the diffusion coefficient and *d*_grad_ the characteristic length of the motility gradient.

Independently of the interaction strength and the size of the colony (if colony size exceeds the characteristic length of the motility gradient) we observe a characteristic length of about 1 *μ*m (see table S2 in the supplementary information). This value is of the order of the size of a single cell and of the average length of the pili. The ratio of the characteristic pili length and the size of the cells affect the interaction range of the pili-mediated cell–cell interactions. The magnitude of the diffusion constant *D*_r_ is strongly affected by the chosen parameters. For weak detachment forces of pili–pili connections *F*_d,sub_, the diffusion coefficient shows a considerable increase (at least one order of magnitude) compared to the parameter set representing strong detachment forces. Thus, cells with weaker interactions are more motile, which may correspond, similarly to the surface-motion of cells (section 3.1) and colonies (section 3.2), to a reduced number of actively pulling pili per cell. A cell possesses less actively pulling pili for a higher detachment probability. To test this idea, we compute how many pili a cell possesses as function of its radial position inside of a colony and how many of those pili are creating a non-zero pulling force (see figure 5(C)). For the chosen parameter sets, we do not observe any change in the total number of pili per cell along the radial position inside the colony, which is almost the same as the maximal number of pili *N*_p_. This results from the fact that the mean life time of the pili is considerably larger than the time scale corresponding to the pili production rate *λ*_p_ = 15 Hz (see figure 5(D)). A more interesting behavior is observed for the number of actively pulling pili per cell, where pili actually generate forces due to retractions. For strong detachment forces *F*_d,pi_, a cell possesses around 14 actively pulling pili, while for weaker detachment forces this number is reduced to 10 pili. In addition, we compute the average life time of the pili of a cell and check how this value depends on the position of the cell within a microcolony (see figure 5(D)). The life time for strong pili–pili interactions increases up to15 s, while it has a value of around 5 s for weak interactions. These results suggest that the large difference in the magnitude of the diffusion coefficients as a function of the strength of pili–pili interactions results from the number of interacting pili and for how long these pili persist.

While we understand differences in the order of magnitude of the diffusion coefficient between different conditions, the microscopic origin of the spatially dependent gradient of motility remains unclear. We find that cells at the surface of a colony possess a smaller number of active pili as compared to cells within the bulk (see figure 5(C)). Furthermore, the fluctuations of the pili number are larger at the surface of microcolonies (see figure 5(E)). This means that on average cells close to the surface have less actively pulling pili, but their number fluctuates stronger than for cells in the bulk of the colonies. These two effects can contribute to an increase in the motility of cells on the surface. Next to their number, the life time of pili (see figure 5(D)) decreases near the surface of the colony. This amplifies the directional fluctuations of pulling events.

In addition to the decrease of active pili bindings and their number fluctuations, a decreasing density of cells towards the surface of the colony also contributes to the motility gradient. In our model, we can calculate the cell number density *ρ*(*d*_com_) as a function of the distance from the center of the colony (see figure 5(F)). We can compare it to the density profile of liquid–liquid or liquid–vapor interfaces, which reads [43, 44]

(10) $$\rho \left(d_{\text{com}}\right)=\frac{\rho_{0}}{2}\cdot \left[1-\text{tanh}\left(\frac{d_{\text{com}}-R_{\text{col}}}{\omega }\right)\right].$$

By fitting this function to density profiles obtained from our model, we can estimate the bulk density *ρ*_0_, the colony radius *R*_col_ and the interface width *ω* [43]. Comparing the values for different parameter sets (see table S3 in the supplementary information), we observe that for the same number of cells and strong interactions, the colony shows a higher bulk density *ρ*_0_ = (0.20 ± 0.01) *μ*m^−3^ and has a smaller radius *R*_col_ = (7.97 ± 0.02) *μ*m, compared to weak interactions with *ρ*_0_ = (0.17 ± 0.02) *μ*m^−3^ and *R*_col_ = (8.19 ± 0.02) *μ*m. We also estimate the width of the interface *ω*, which has a value of (0.16 ± 0.04) *μ*m for strong pili–pili-interactions and (0.41 ± 0.06) *μ*m for weak pili–pili-interactions (see table S3 in the supplementary information). Thus, weaker interactions increase the interfacial width of the cell densities. Altogether, the higher density reduces the motility of the cells by reducing the volume in which a cell can move freely. The pronounced peak observed for cells on the surface of microcolonies characterized by the strong parameter set originates from the nematic order of the diplococcus-shaped cells close to the colony surface (see figure 5(F)). To determine the nematic order parameter, we computed the angle *α* between the axis connecting the two cocci and the vector pointing from the center of the colony to the cell position. The nematic order parameter is given by [45]:

(11) $$S=\langle \frac{3\cdot {\text{cos}}^{2}\alpha -1}{2}\rangle .$$

We observe an overall random distribution of cell directions inside of the colony, with a nematic order parameter *S* close to 0 and a bias towards a tangential orientation of cells close to the surface with *S* < 0 (see figure 5(H)). This bias results from the purely attractive nature of the pili-mediated forces: a cell favors orientations which maximize the number of pili–pili interactions. Within a microcolony, the distribution of cells is isotropic and thus no preferred cell orientation exists that maximizes the number of attached pili. However, this is different at the surface of the microcolony where cells can align tangentially to the colony surface to increase their number of pili bindings. In summary, a colony characterized by the strong parameter set is very dense and has only a small interfacial width, where cells tend to align parallel to the surface.

In order to test if a solid-like behavior of bulk cells might be the reason for the observed oscillations in the cell number density (see figure 5(F)), we computed the radial pair-correlation function *g*(*r*). If the colony possesses solid-like properties, we would expect to see distinct correlations between the cell positions for strong interactions and less pronounced correlations for weaker interactions. Instead, we observe an almost identical shape of the pair correlation function for the two selected parameter sets (see figure 5(G)) with correlations only reaching up to 3–4 *μ*m, corresponding roughly to the next neighbors distance. Therefore, the colonies still exhibit liquid-like behavior. This observation is consistent with a diffusive behavior of the mean-squared displacements of the cell trajectories within microcolonies (see figure S2 in the supplementary information).

Ultimately, all of the processes described above are linked to the distribution of forces generated by the attached pili. In our model, every cell is subjected to friction, pili-mediated and excluded volume forces which strictly balance each other. The motion of the cells is thus caused by the sum of the pili-mediated and the excluded volume forces, which we refer to as net force. We can split the net force acting on each cell into a component normal and tangential to the surface of the colonies. The mean values of these forces are always close to zero (see figures S3(B) and (C) in the supplementary information), due to the balance of the repulsive excluded volume forces and the attractive pili–pili forces. Remarkably, the standard deviation of the net forces (see figures 5(H) and (I)) exhibits a pronounced gradient for a wide range of parameters, with larger values at the surface of the colonies. This observation is most likely connected to the increased fluctuations of the number of attached pili close to the surface for both parameter sets and the decreased number of actively pulling pili.

The most important observation characterizing the internal dynamics of *N. gonorrhoeae* microcolonies is the existence of a gradient of motility in which cells on the surface are more motile than cells in the bulk of a microcolony. We found that this gradient correlates with gradients in the number of actively pulling pili and forces that are acting on the cell. The interplay of all these effects contributes to the appearance of the motility gradient.

For the case of smaller pili–pili detachment forces *F*_d,pil_ = 50 pN (see [27, 29]), the cells within a colony are more motile than for the weak parameter set (see figure S5). Additionally, we checked how different values of *k*_cc_ affect the properties of the microcolonies and observe that for a broad range of values the outcome is not affected (see figure S7).

### 3.4. Coalescence of two microcolonies

Up to now we have only considered individual cells of large bacterial colonies leaving aside the questions of how these colonies form and grow. In *N. gonorrhoeae*, the growth of microcolonies is driven mostly by bacterial self-assembly, while the proliferation of cells contributes only very little to growth [19]. An important step during the formation of a larger microcolony is the coalescence of two individual microcolonies of smaller size [19]. This process includes rearrangements of cells within the merging colonies and is thus highly affected by the internal properties of microcolonies, as described in 3.3.

**Figure 6.**
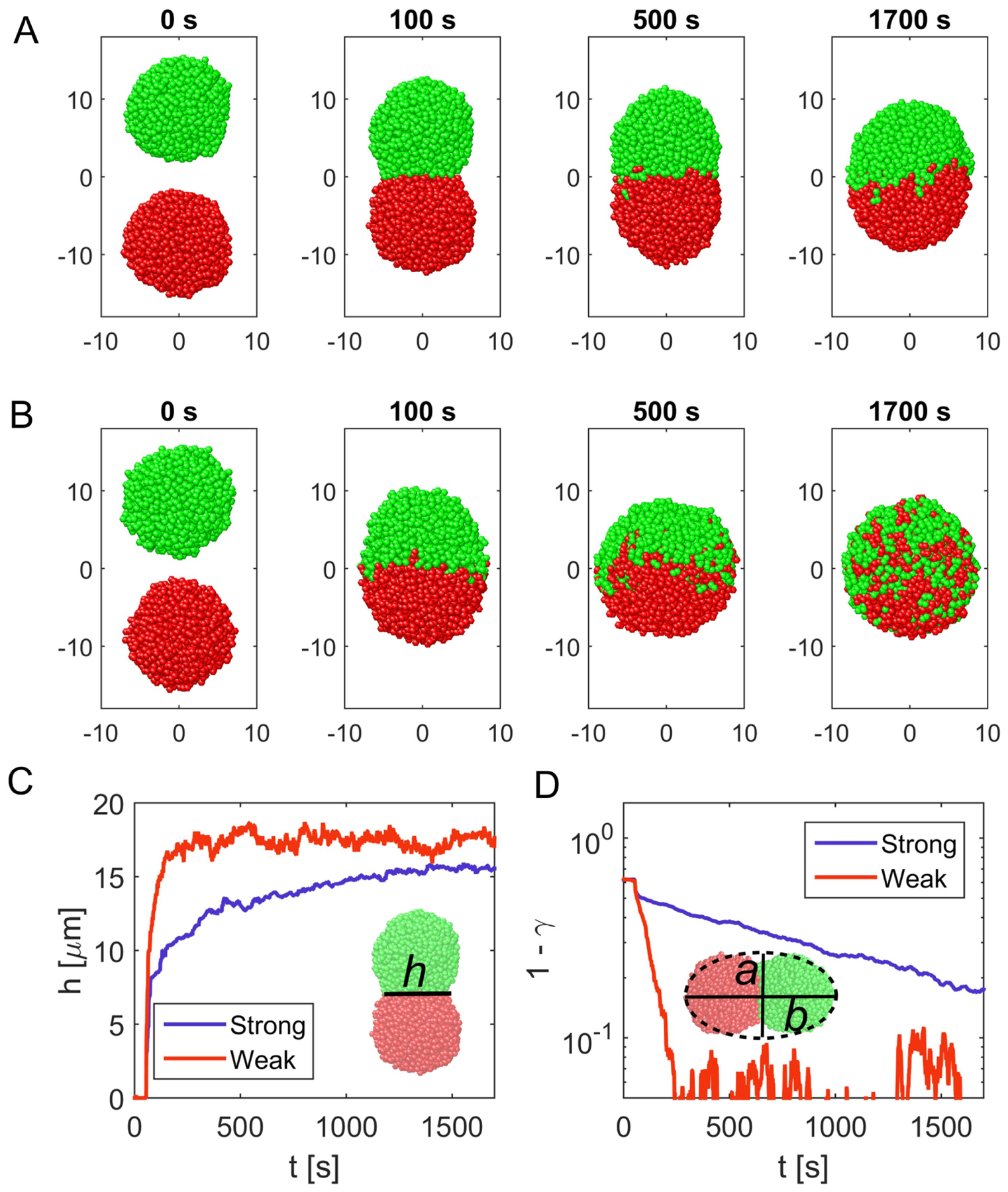
Coalescence of two microcolonies. (A) Merging dynamics of two microcolonies consisting of 1000 cells each for the parameter set with strong pili–pili interactions (see table 3). (B) Merging dynamics corresponding to the parameter set with weak pili–pili interactions (see table 3). (C) Height *h* of the bridge forming between the two colonies as a function of time for strong interactions (blue) and weak pili–pili interactions (red). For weaker interactions, individual cells are more motile and the closure of the bridge takes a few seconds. The inset depicts the definition of the bridge *h*. (D) Axis ratio *γ* = *a/b* of the short *a* and the long axis *b* for an ellipse fitted to the envelope of the two-dimensional projection of the colony as a function of time for strong interactions (blue) and weak interactions (red). For a spherical shape the ratio has a value of 1. Thus, the difference 1 – *γ* relaxes towards 0 and exhibits an exponential behavior. The inset sketches the ellipse fitted to the cross-section of the colony.

To get a deeper understanding of the processes that drive the merging in our model, we studied the coalescence of two *in silico* colonies (see figures 6(A) and (B)) consisting of 1000 cells each. During the coalescence, the contact area that forms between the two colonies is called a bridge (see figure 6(C)), in analogy to a liquid bridge of coalescing liquid droplets. We observe that the initial closure of the bridge occurs within the first few seconds, which is the time required for the pili to pull colonies together (see figures 6(A)–(C)). In order to quantify this behavior, we measured the bridge height, the axial symmetric diameter of the bridge (see figure 6(C) and section S5.4 in the supplementary information).

**Figure 7.**
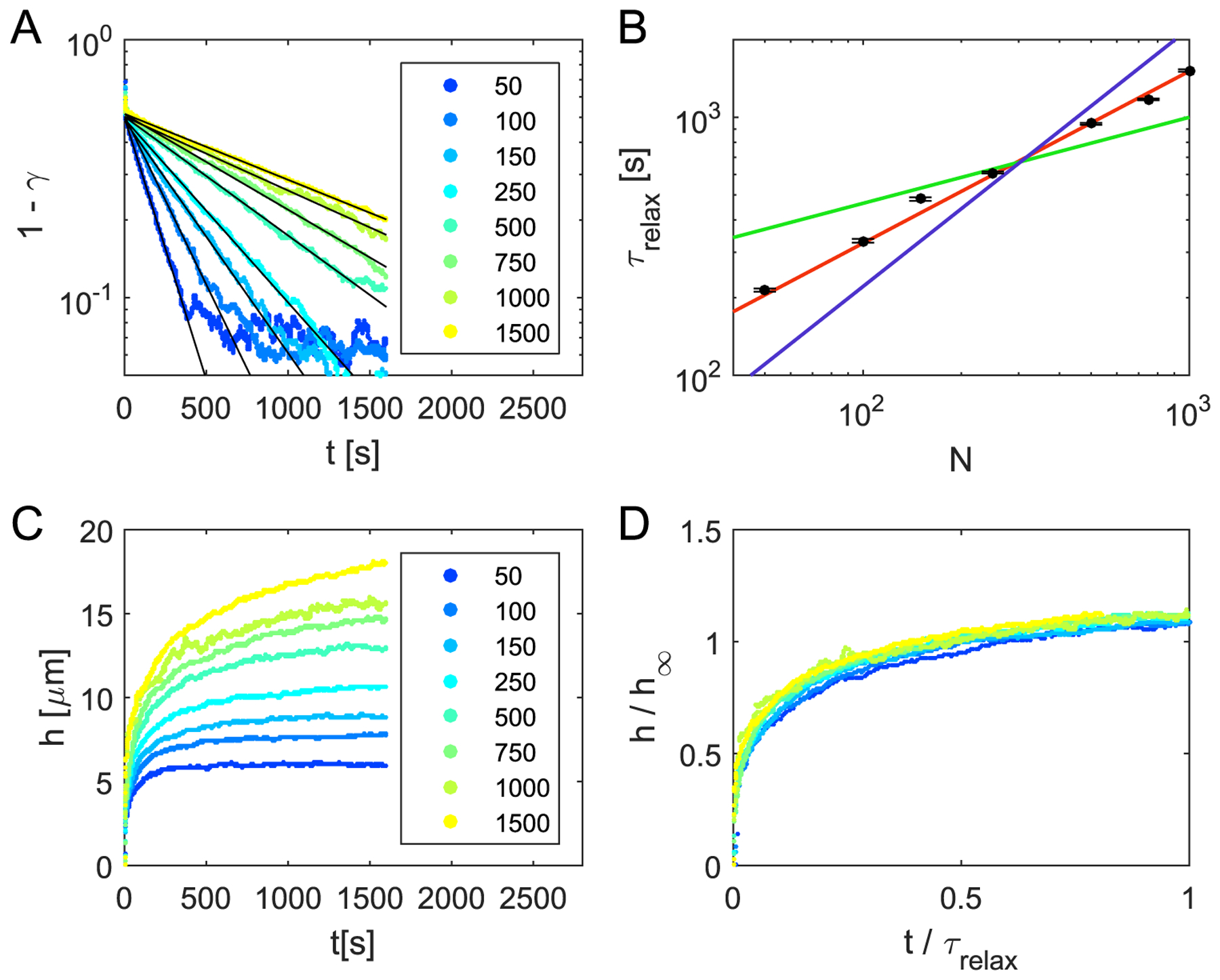
Scaling of the merging dynamics. (A) Ratio *γ* of the short and long axis of the ellipse as a function of time for the coalescence of differently sized colonies. The legend shows the numbers of cells in the individual colonies. By fitting an exponential function (black lines) one can extract the relaxation time *τ*_relax_ as a function of the cell number *N*. (B) The black dots show the relaxation times resulting from the exponential fit of the axis ratio as a function of the cell number. While *τ*_relax_ increases with time, we tested different scalings: *τ*_relax_ ∝ *N*^1/3^(green), *τ*_relax_ ∝ *N*^2/3^ (red) and *τ*_relax_ ∝ *N*^1^ (blue). The extracted values for *τ*_relax_ seem to agree best with *τ*_relax_ ∝ *N*^2/3^. This suggest that the relaxation time is proportional to the surface area of the colonies. (C) Bridge heights *h* as a function of time for different number of cells inside the colony. The legend shows the numbers of cells in the individual colonies. (D) Rescaled bridge height: the height of the bridge is rescaled by *h*_∞_ = 1.4 · *N*^1/3^ *μ*m and time by the relaxation time *τ*_relax_ = 15.1 · *N*^2/3^ s. The relaxation time is obtained from 1 – *γ* (*t*) (see (A) and (B)). The rescaling for the height *h*_∞_ corresponds to the diameter of a spherical colony which is reached at times much larger than the relaxation time *τ*_relax_ (see figure S4 in the supplementary information). After rescaling all curves collapse.

In addition to the closure of the bridge, we also study the relaxation of the microcolonies towards a spherical shape. For this, we fit an ellipse to the cross section of the microcolony and compute the ratio *γ* of its shorter and longer axis (see section S5.4 in the supplementary information and figure 6(D)). We suggest that the relaxation processes are determined by the ‘two-component’ nature of the colony with more motile cells on the surface and less motile cells in the bulk. We thus expect that the relaxation times would be sensitive to the overall size of the colony, by which we alternate the surface to volume ratio. To test this hypothesis, we modeled the merging of two colonies, each consisting of an equal number of cells ranging from 50 to 1500 cells. Here, we only consider strong pili–pili interactions. The relaxation of the axis ratio *γ* towards 1 can be described by an exponential function with the characteristic time *τ*_relax_ (see figure 7(A)). We find that the relaxation time as a function of the number of cells in one of the initial colonies follows the scaling $\tau_{\text{relax }}\propto N^{\frac{2}{3}}$ (see figure 7(B)). The volume of the colony is proportional to the number of cells in a colony, *N* ∝ *R*^3^ (see figure S4 in the supplementary information) and thus for the relaxation time *τ*_relax_ ∝ *R*^2^. Hence, the relaxation time is proportional to the surface area of the colonies. We can use this result to check the scaling of the bridge height as a function of time during coalescence. Therefore, we compute the bridge height as a function of time for different colony sizes and rescale the time by the characteristic relaxation time proportional to the surface area *R*^2^. Additionally, we normalize the bridge to the diameter of the the approaching sphere *h*_∞_ (see figure S4 in the supplementary information). Indeed, using these scalings we find that the relaxation curves collapse (see figures 7(C) and (D)).

We can compare our results to the coalescence of liquid droplets. Coalescence of liquid droplets is driven by surface tension and thus bridge closure and relaxation to a sphere occur within similar times [46]. However, we observe that for the experimentally relevant parameters, the merging of *N. gonorrhoeae* colonies exhibits two distinct relaxation regimes [19]. Initially, the two colonies approach each other and close the bridge. This process is followed by a slower relaxation of the resulting ellipsoid towards a spherical shape. For the case of very small pili–pili detachment forces *F*_d,pil_ = 50 pN, we see that it is no longer possible to find two distinct time scales (see figure S6) and the coalescence proceeds in a more liquid-like manner.

### 3.5. Self-assembly of microcolonies on a surface

**Figure 8.**
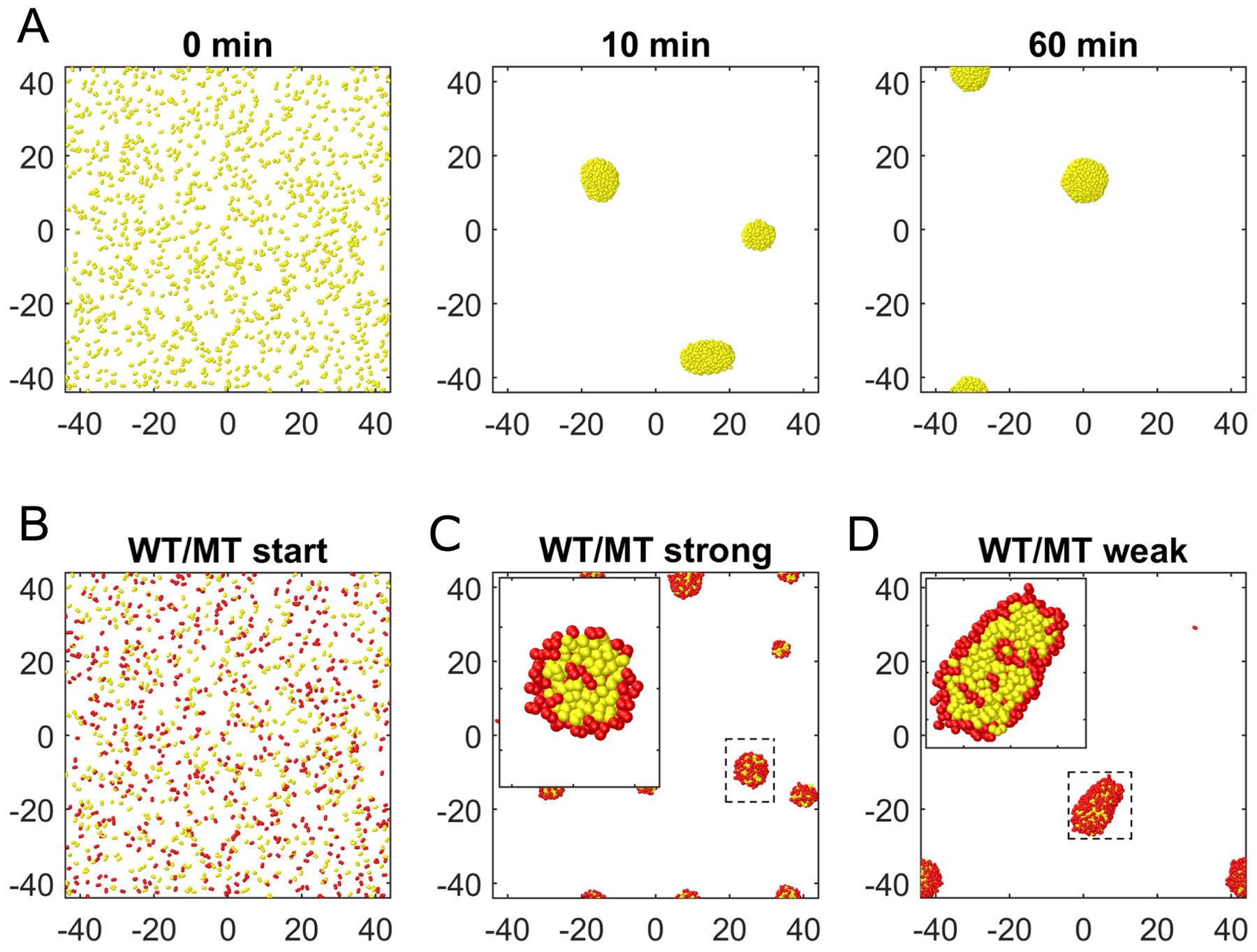
Assembly of microcolonies driven by pili-mediated cell-to-cell interactions. (A) Assembly of 1200 cells on a substrate with weak pili-substrate interactions and strong pili–pili-interactions (see tables 3 and 4). After initializing cells homogeneously on the substrate (top left), colonies begin to form after a few minutes (top center). They grow by single cells colliding with the less motile colonies. After one hour, almost all cells are assembled into colonies (top right). (B) Initial mixture of normal cells (yellow) and ΔpilT mutants (red). These mutants have pili which cannot pull. (C), (D) Colonies formed after one hour for strong and weak pili-surface interactions. Stronger pili-surface interactions lead to smaller colonies. The corresponding initial state is given in (B). The inset depicts a close-up in of a typical colony and shows that the mutant cells accumulate a the surface of the colony.

**Figure 9.**
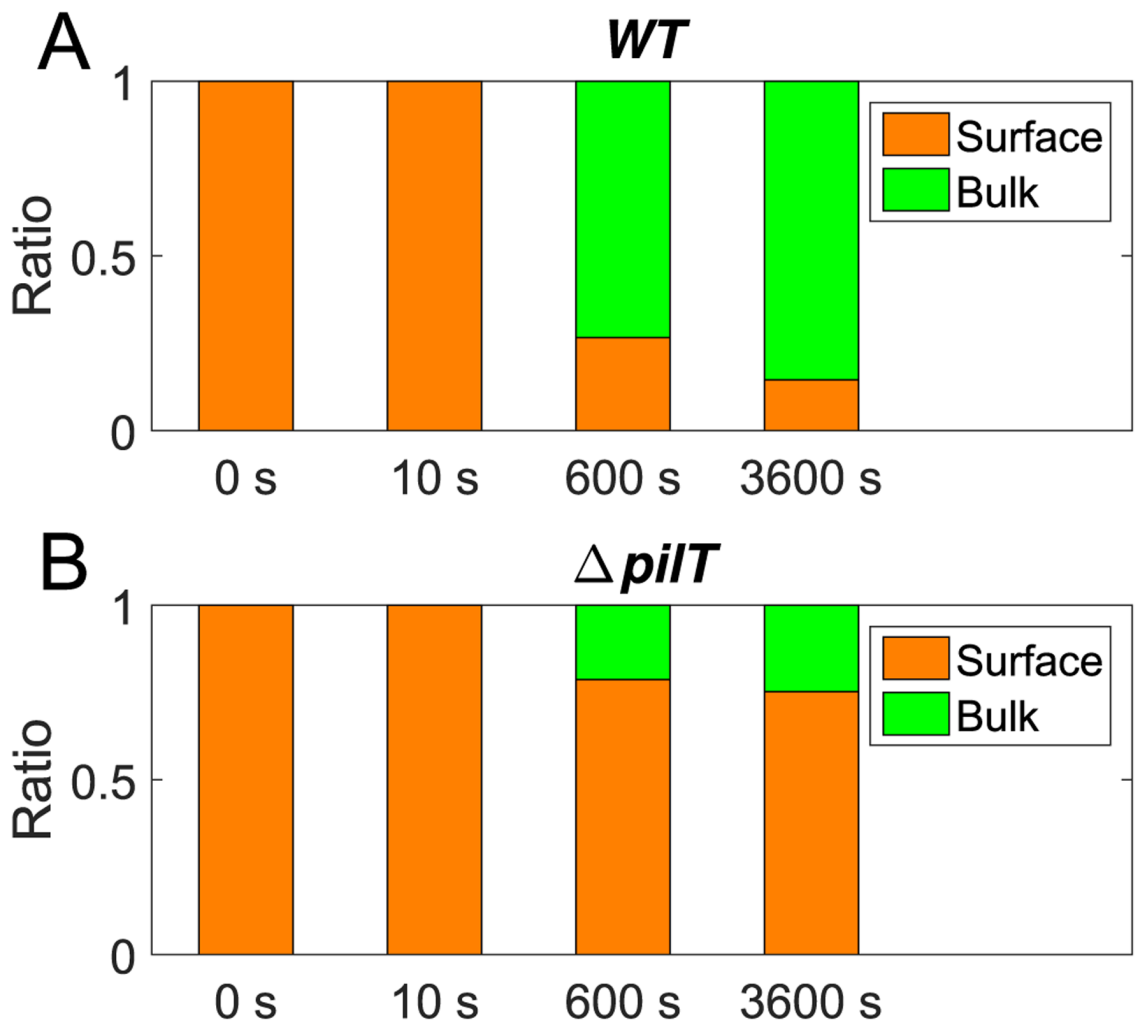
Ratio of surface and bulk cells for *in silico* wild-type and ΔpilT cells. Initially 1200 individual cells are randomly distributed on a substrate and will form microcolonies within minutes. We mixed 600 wild-type and 600 ΔpilT mutants with non-retracting pili and studied the amount of cells on the surface and within the bulk for both species. (A) Ratio of *in silico* wild-type (WT) cells at the surface (orange) and inside of the colony (green). Surface cells are identified by computation of the alpha shape, see section S5.5 in the supplementary information. The colonies form such that within 10 minutes, the wild-type cells can be found preferentially inside of the colonies. (B) Ratio of mutant cells identified as surface (orange) and bulk (green) cells. A larger fraction of ΔpilT mutants can be found on the surface of the colonies.

The surface-motility of individual cells and microcolonies, the internal dynamics of colonies and their coalescence all play important roles during the assembly of individual cells into multiple microcolonies on a substrate. Our computational model allows to combine all these contributions to study the process of self-assembly of microcolonies (see figure 8(A)). Importantly, we can now ask how the assembly process is affected by altering the dynamics of pili. What happens if pili are not able to generate forces? Obviously, cells with such pili would not be able to move and assemble colonies. We can, however, consider a mixture of a mutant (particularly an existing ΔpilT mutant cell for which the motor is unable to retract the pilus) and the wild-type cells in 1:1 proportion. Remarkably, we observe that the non-pulling cells are incorporated into the colonies, however these cells accumulate at the surface of the microcolony (see figures 8(B)–(D) and 9).

We can quantify, by computing the alpha shape (see section S5.5 in the supplementary information) of the cocci positions of the sum of wild-type and mutant cells, which cells belong to the surface of the colony and which cells belong to its bulk. By comparing the ratio of bulk cells versus surface cells for the wild-type and the mutant cells, we confirm our observation quantitatively (see figure 9) and can relate it to previous experimental data [29]. The demixing of cells with different pulling forces is consistent with the differential adhesion hypothesis, which explains the separation of two different cell populations in an agglomerate of cells based on a difference in the adhesive interactions of the cells [47–49]. However, in our system there is no difference in the passive adhesive properties of cells, but instead our bacterial cells differ in their ability to retract their pili and thus there is a difference in active force generation.

## 4. Discussion

In this work, we presented, to our knowledge, the first computational model of microcolonies consisting of single cells that are interacting mechanically via individual pili. Within this model, we computed the forces originating from the pulling of pili attached to a surface and to pili of other cells. The pili-mediated force generation drives a wide range of processes relevant to *N. gonorrhoeae* bacteria, ranging from the surface-motion of single cells and colonies to the formation of larger colonies due to interactions of smaller ones. Our findings might have implications for a better understanding of the gonorrhea infection, as the microcolonies of *N. gonorrhoeae* are also the infectious units of the disease. By proposing this quantitative model of early biofilm formation in *N. gonorrhoeae*, we can gain new insights into how to better control the formation of colonies, for example by altering the interactions of pili with the substrate or other pili. This model can be modified to encompass other bacterial shapes or pili characteristics, for example those of *Pseudomonas aeruginosa*, *Neisseria meningitidis* or *Neisseria elongata* bacteria. We also see several directions in which the model and its implementation can be further extended. Allowing for multiple pili intersections, pili bundles and confining pili growth to intercellular volume are the directions of our future work.

## Supplementary Material

Supplementary Material

## Figures and Tables

**Table 1.** Fixed parameters for the simulations. The choice of parameters is motivated by the corresponding references.

Parameter	Value	Reference
Cocci radius *R*	0.5 *μ*m	[22]
Cocci distance *d*	0.6 *μ*m	[22]
Cell-cell excl. vol. const. *k*_*cc*_	2 × 10^4^ pN *μ*m^−1^	
Cell-sub excl. vol. const. *k*_cs_	4 × 10^4^ pN *μ*m^−1^	
Translational mobility *μ*_trans_	1 mm (s pN)^−1^	
Rotational mobility *μ*_rot_	2 (s pN)^−1^	
Pili persistence length *l*_p_	5 *μ*m	[26]
Pili production rate λ_p_	15 Hz	[23]
Maximal pili number N_pili_	15	[28]
Pili protrusion velocity *v*_pro_	2 *μ*m s^−1^	[22,23]
Pili retraction velocity *v*_ret_	2 *μ*m s^−1^	[22,23]
Pili retraction rate λ_ret_	1.33 Hz	[22]
Pili spring constant *k*_pull_	2000 pN *μ*m^−1^	[33]

**Table 2.** Parameters which were sampled.

Parameter
Pili pili attachment rate λ_pil_ (Hz)
Pili substrate attachment rate λ_sub_ (Hz)
Pili substrate detachment force *F*_d,sub_ (pN)
Pili substrate detachment time *τ*_d,sub_ (s)
Pili pili detachment force *F*_d,pil_ (pN)
Pili pili detachment time *τ*_d,pil_ (s)

**Table 3.** Parameters of the strong and weak sets characterizing the strength of pili–pili-interactions.

Parameter	Weak	Strong
Pili pili detachment force *F*_d,pil_ (pN)	120	360
Pili pili detachment time *τ*_d,pil_ (s)	50	50

**Table 4.** Parameters of the strong and weak sets characterizing the strength of pili-substrate-interactions.

Parameter	Weak	Strong
Pili substrate detachment force *F*_d,sub_ (pN)	180	300
Pili substrate detachment time *τ*_d,sub_ (s)	10	30
