# Supplementary material for: Multiscale modeling of bacterial colonies: how pili mediate the dynamics of single cells and cellular aggregates
Source: New J Phys. Author manuscript; Available in PMC 2021 May 19. (PMC8132470; doi:10.1088/1367-2630/aa5483)
Supplement: Supplementary Material [file NIHMS1552593-supplement-Supplementary_Material.pdf]

Multiscale Modeling of bacterial colonies: How pili mediate the dynamics of single cells and aggregates - Supplementary Information

Wolfram Pönisch[1], Christoph A. Weber[1,2], Guido Juckeland[3], Nicolas Biais[4,5] and Vasily Zaburdaev[1]

[1]Max Planck Institute for the Physics of Complex Systems, 01187 Dresden, Germany
[2]Paulson School of Engineering and Applied Sciences, Harvard University, Cambridge, MA 02138, USA
[3]Department of Information Services and Computing (FWC), Helmholtz-Zentrum Dresden-Rossendorf e.V, 01314 Dresden, Germany
[4]Department of Biology, Brooklyn College, City University of New York, Brooklyn, New York 11210, USA
[5]Graduate Center of CUNY, New York 10016, USA

E-mail: `vzaburd@pks.mpg.de`

Contents

S1 Supplemental Figures **3**

S2 Additional details of the computational model **10**

S3 Fitting Results **13**
S3.1 Velocity autocorrelation of single cells on a surface . . . 13
S3.2 Profile of diffusion coefficient of cells inside of colony . . . 13
S3.3 Profile of cell densities of a spherical colony . . . 13

S4 Simulation Details **15**
S4.1 Single Cells . . . 15
S4.2 Single Colony on Substrate . . . 15
S4.3 Free Single Colony . . . 16
S4.4 Colony Coalescence . . . 16
S4.5 Assembly on a substrate . . . 17

S5 Data Analysis **18**
S5.1 Single Cells . . . 18
S5.2 Single Colony on Substrate . . . 18
S5.3 Free Single Colony . . . 18
S5.4 Colony Coalescence . . . 19
S5.5 Assembly on a substrate . . . 19

S6 References **20**

S1. Supplemental Figures

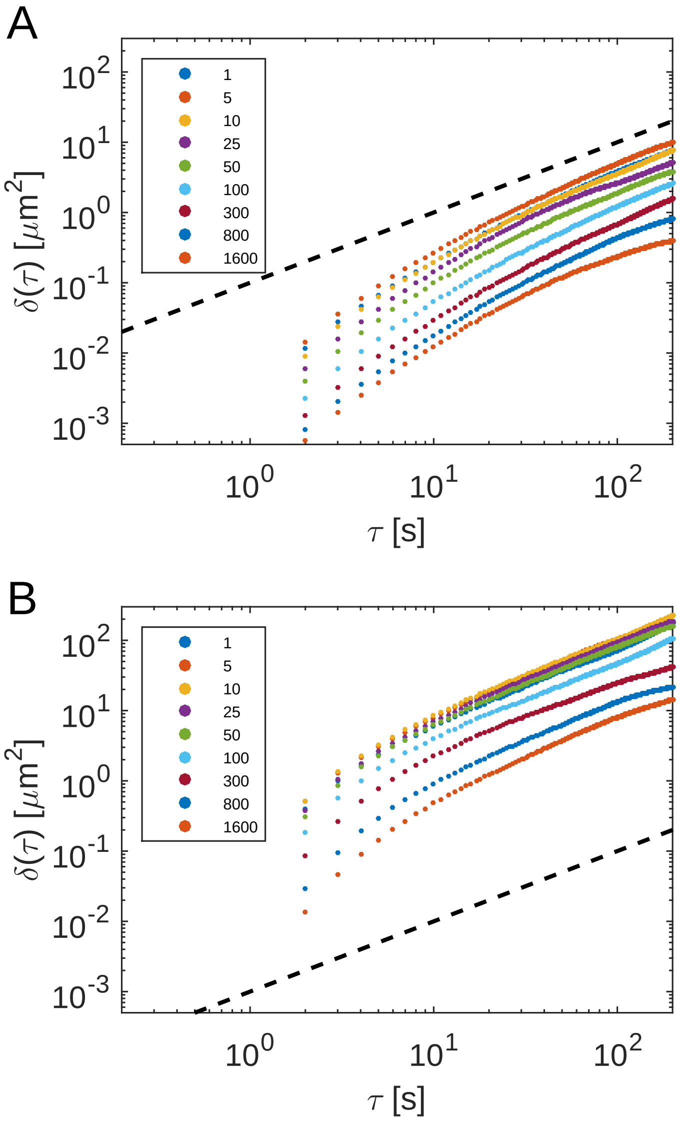

Figure S1. Mean squared displacement (MSD) of single colonies on a substrate. The weak and strong parameter sets are defined in table 4. (A) MSD of differently sized colonies for a parameter set characterizing a strong attachment to the substrate. The black dashed line represents a linear time-dependence. Thus for large time steps τ the cells behave diffusively. (B) MSD for the case of a weak attachment to the substrate.

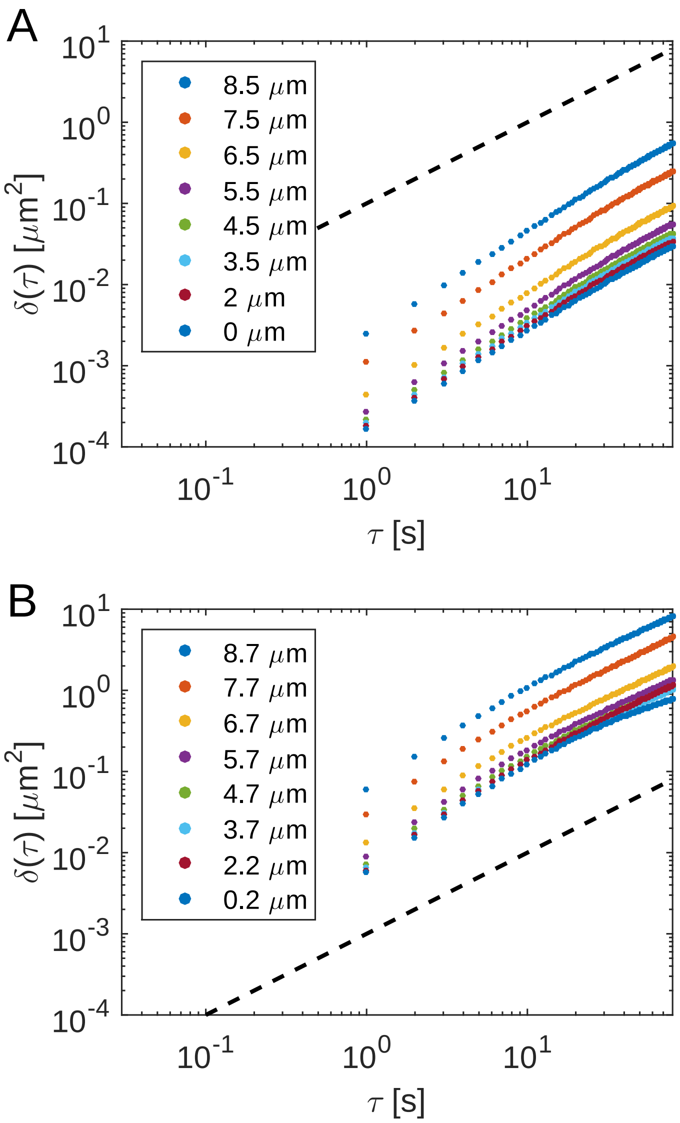

Figure S2. MSD of individual cells within colonies consisting of 1700 cells for different mean distances from the center of the colony. The weak and strong parameter sets are defined in table 3. (A) MSD for strong pili-pili-interactions. The black dashed line represents a diffusive scaling, $\delta(\tau) \propto \tau$. (B) Mean squared displacement for weak pili-pili-interactions. Again, black line represents a diffusive scaling, $\delta(\tau) \propto \tau$.

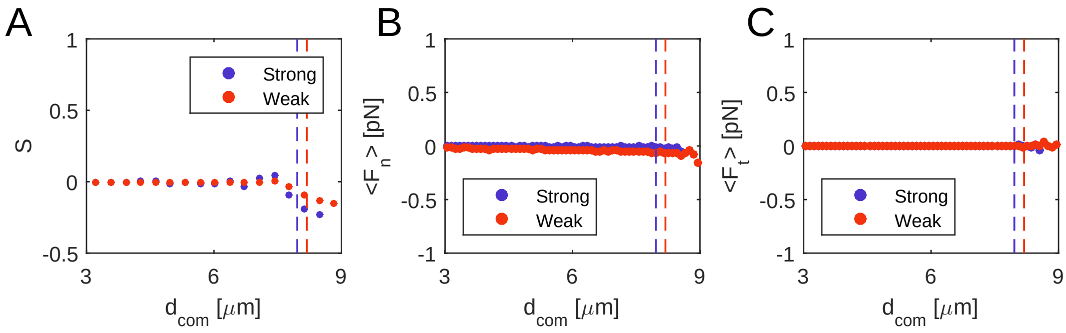

Figure S3. Properties of individual cells within microcolonies. The vertical lines in (A-C) highlight the radius of the microcolonies as computed from the distribution of the cell number density. (A) Nematic order parameter S (see equation (12)) of cells within a colony. Here we use the orientation of a cell which is defined its long axis and compute the nematic order relative to the radial vector pointing towards the cell position. The case $S = 0$ indicates a random distribution of orientations, while $S < 0$ corresponds to a tangential orientation of the cell relative to the colony surface. This effect is more pronounced for the strong parameter set, characterized by a larger detachment force $F_{\mathrm{d,pil}}$. (B,C) Mean of the normal and tangential net forces acting on a cell relative to the surface of the colony. The attractive pili-pili interactions and the repulsive excluded volume interactions balance each other such that the mean net force acting on a cell vanishes. The weak (blue) and strong (red) parameter sets are defined in table 3.

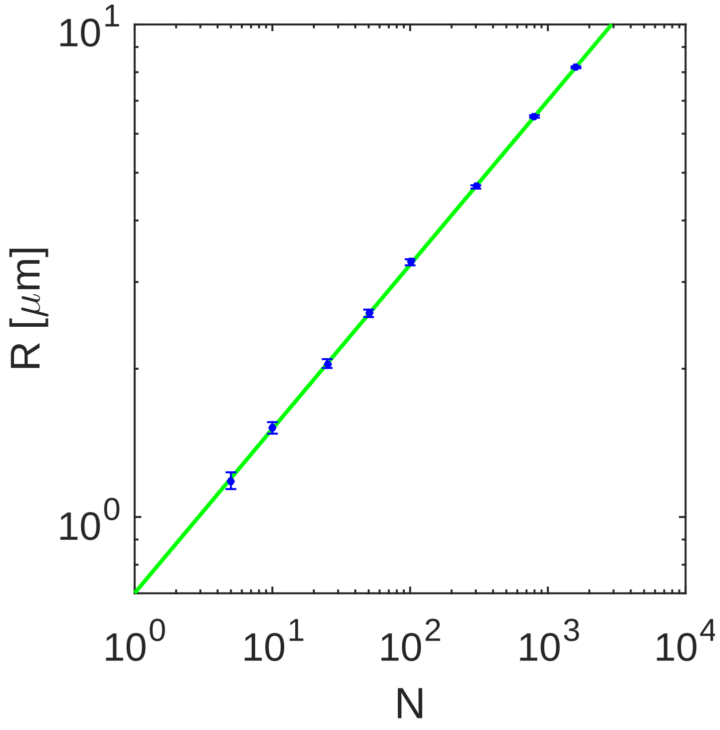

Figure S4. Dependence of the colony radius on the cell number (blue dots) for a colony without substrate interactions. Here the colony radius corresponds to the radius of a circle having the same area as the envelope of the two-dimensional projection of a colony. The green curve is $R = 0.7 \cdot N^{1/3}$ μm. Thus the volume is proportional to the cell number.

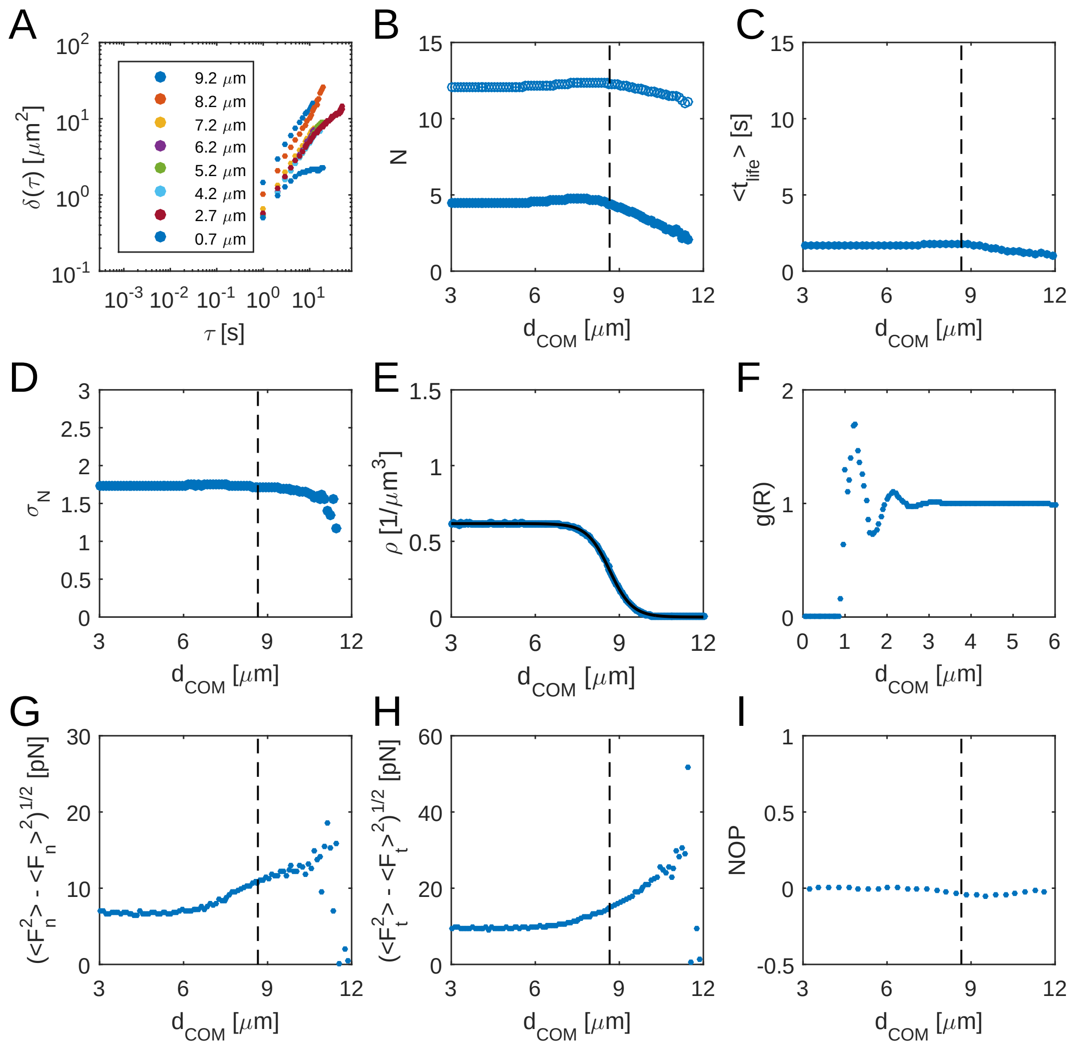

Figure S5. Internal properties of individual colonies for $F_{\text{stall}} = 180$ pN, $F_{\text{d,pil}} = 50$ pN and $\tau_{\text{d,pil}} = 50$ s. Vertical dashed lines represent the corresponding radii of the colonies R_{col} by fitting the density profile of cells within the microcolony according to equation (10). For such a small detachment force the cells are highly motile. (A) Mean-Squared displacements of cells as a function of their distance d_{com} from the center of the colony. We see that cells do not stay longer than 30-60 seconds in their shell, thus it is not possible to estimate a long time limit diffusion coefficient. (B) Mean of the number of all pili (empty circles), and of the pili which generate a pulling force on cells (filled circles) as a function of d_{com}. (C) Mean life time of the pili of cells within a microcolony. Pili have a life time which corresponds to the life time of a free pilus $t_{\text{life}}^{(\text{free})} = l_{\text{ch}}/v_{\text{ret}} + l_{\text{ch}}/v_{\text{pro}} = 1.5$ s. (D) Standard deviation of the number of all pili, and of the pili which generate a pulling force on cells as a function of d_{com}. (E) Cell number density ρ of cells as a function of the distance d_{com} from the center of the colony. The lines represent fits with a tanh-function, which is also a solution of the interfacial profiles in phase separated binary mixtures [1]. (F) Pair-correlation function of the centers of cells within a colony. The first peak consists of two individual peaks resulting from the diplococcus shape of the cells. (G) Standard deviation of the normal net forces acting on a cell relative to the surface of the colony. (H) Standard deviation of the tangential net forces acting on a cell relative to the surface of the colony. (I) Nematic order parameter S (see equation (12)) of cells within a colony.

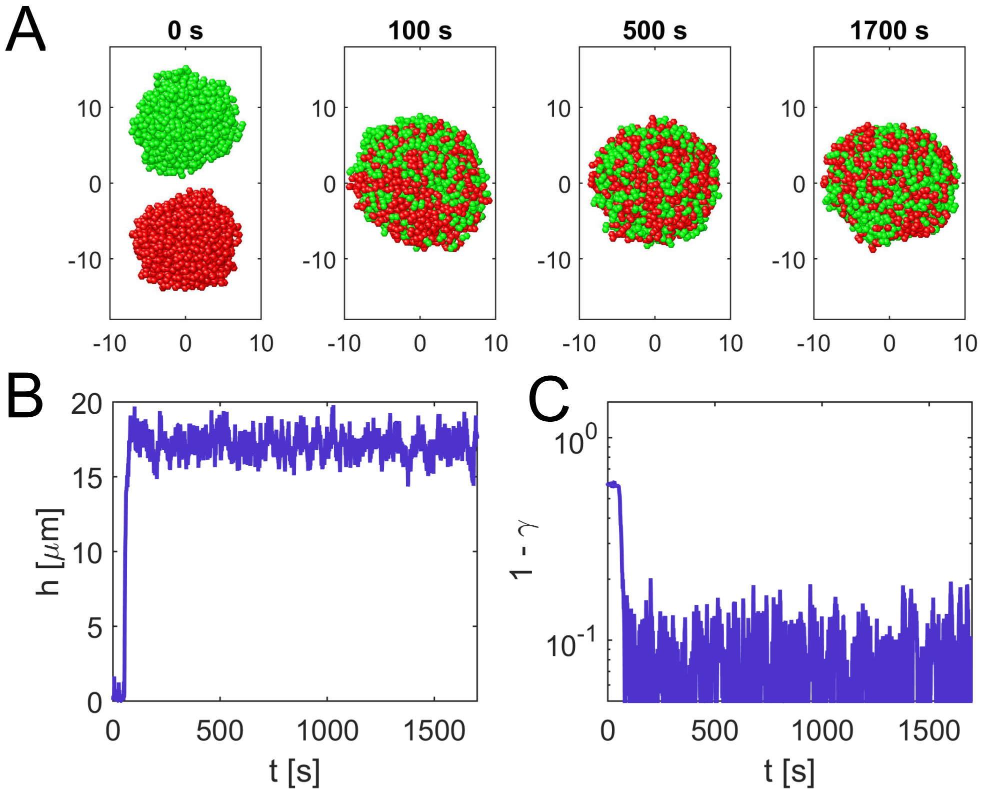

Figure S6. Coalescence of two microcolonies for $F_{\text{stall}} = 180$ pN, $F_{\text{d,pil}} = 50$ pN and $\tau_{\text{d,pil}} = 50$ s. The two colonies merge fully within seconds. (A) Merging dynamics of two microcolonies consisting of 750 cells. (B) Height h of the bridge forming between the two colonies. (D) Axis ratio $\gamma = a/b$ of the short a and the long axis b for an ellipse fitted to the envelope of the two-dimensional projection of the colony as a function of time.

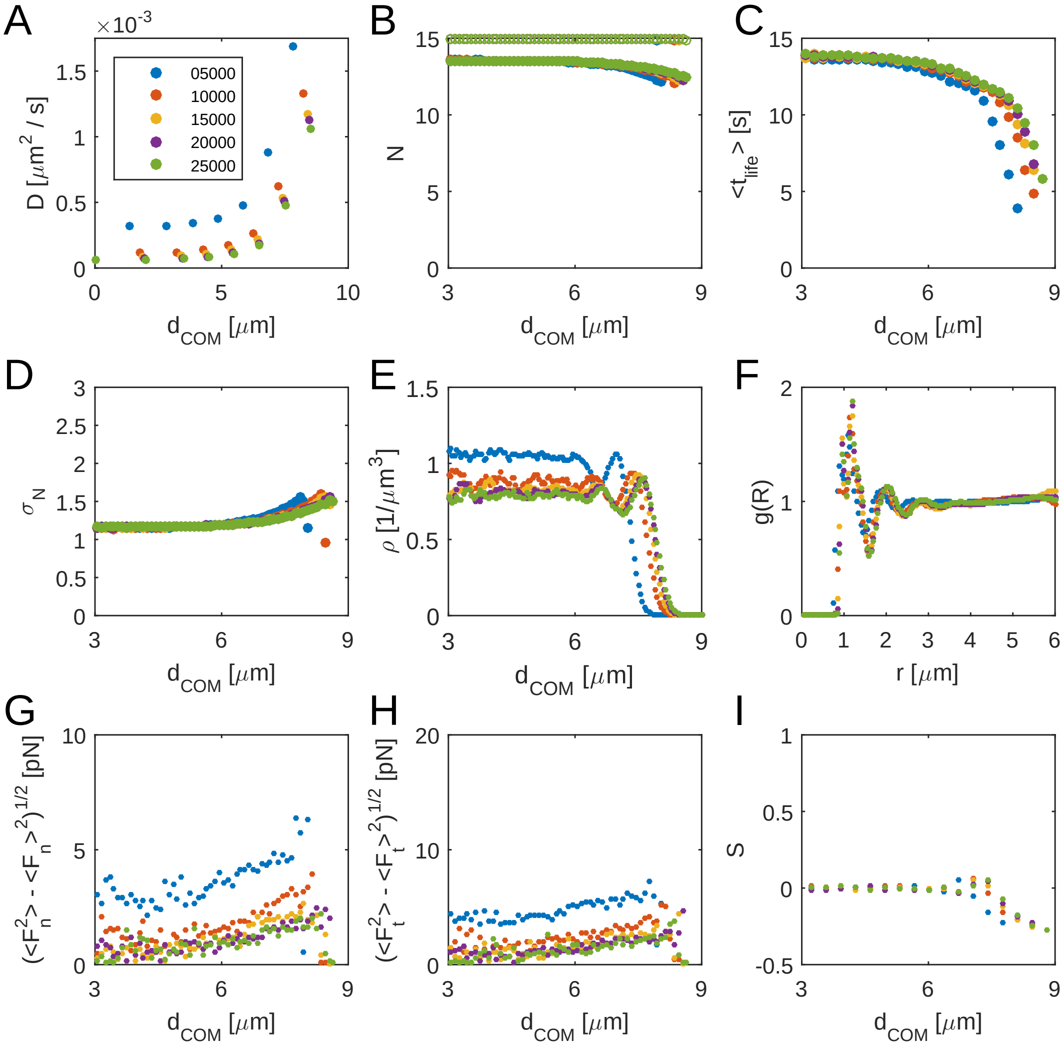

Figure S7. Internal properties of individual colonies for strong pili-pili-interactions (see table 3) and different values of $k_{cc} = 5000,\ 10000,\ 15000,\ 20000,\ 25000$ pN/μm. We see that the results are almost independent of the excluded volume constant for values larger than 10000 pN/μm. For softer cells the intersections of the cells are too strong and affect the result. (A) Diffusion coefficient as a function of the distance $d_{\rm com}$ from the center of the colony for an individual cell. (B) Mean of the number of all pili, and of the pili which generate a pulling force on cells as a function of $d_{\rm com}$. (C) Mean life time of the pili of cells within a microcolony. (D) Standard deviation of the number of all pili, and of the pili which generate a pulling force on cells as a function of $d_{\rm com}$. (E) Cell number density ρ of cells as a function of the distance $d_{\rm com}$ from the center of the colony. (F) Pair-correlation function of the centers of cells within a colony. (G) Standard deviation of the normal net forces acting on a cell relative to the surface of the colony. (H) Standard deviation of the tangential net forces acting on a cell relative to the surface of the colony. (I) Nematic order parameter S (see equation (12)) of cells within a colony. Here we use the orientation of a cell which is defined its long axis and compute the nematic order relative to the radial vector pointing towards the cell position. The case $S = 0$ indicates a random distribution of orientations, while $S < 0$ corresponds to a tangential orientation of the cell relative to the colony surface.

S2. Additional details of the computational model

Geometry of an individual cell and free pili dynamics

A pilus k is characterized by two points: its start $\mathbf{x}_k^{(\mathrm{s})}$ and end point $\mathbf{x}_k^{(\mathrm{e})}$. The contour length of a pilus is defined to be $l_k^{(\mathrm{con})} = \left|\mathbf{x}_k^{(\mathrm{e})} - \mathbf{x}_k^{(\mathrm{s})}\right|$. New pili are produced with a rate λ_p until a cell has a maximal number of pili, N_p. In our simulations we do not check for the production of new pili for every time step, but only every $\Delta t_\mathrm{d} = 0.02$ s. This simplification allows to reduce the numerical cost of our simulations and is valid as long as the time corresponding to the attachment rates is much larger. The start point of the pilus k is randomly distributed on the surface of one of the two cocci ($\mathbf{r}_i^{(\mathrm{a})}$ and $\mathbf{r}_i^{(\mathrm{b})}$) of the cell i, with the constraints that the start point cannot be inside of any cocci, ensured by the condition $\left|\mathbf{r}_i^{(\mathrm{a})} - \mathbf{x}_k^{(\mathrm{s})}\right| \geq R$ and $\left|\mathbf{r}_i^{(\mathrm{b})} - \mathbf{x}_k^{(\mathrm{s})}\right| \geq R$ with the cocci radius R. A new pilus k protrudes from the surface of coccus of cell i with a velocity v_pro. For the position of the pilus end point it follows

$$\mathbf{x}_k^{(\mathrm{e})} = \mathbf{x}_k^{(\mathrm{s})} + l_k^{(\mathrm{fr})} \frac{\mathbf{x}_k^{(\mathrm{s})} - \mathbf{r}_i^{(\mathrm{j})}}{\left|\mathbf{x}_k^{(\mathrm{s})} - \mathbf{r}_i^{(\mathrm{j})}\right|}, \tag{S1}$$

where $l_k^{(\mathrm{fr})}$ is the free length of a pilus and $j = a, b$, giving the position of the coccus from which the pilus emerges. The free length is defined as the length of the pilus if there is no force acting on the polymer. The change of the free length of a protruding pilus k is given by

$$l_k^{(\mathrm{fr})}(t + \Delta t) - l_k^{(\mathrm{fr})}(t) = v_\mathrm{pro} \Delta t. \tag{S2}$$

The pili switch to the retraction state with a probability that corresponds to a rate λ_ret. Each pilus retracts with the velocity v_ret, which is related to the free pili length by

$$l_k^{(\mathrm{fr})}(t + \Delta t) - l_k^{(\mathrm{fr})}(t) = -v_\mathrm{ret} \Delta t. \tag{S3}$$

If the end point of a perpendicularly protruding or retracting pilus is inside of the substrate (see figure 1, main text), the end point will slide along the substrate. While the contour length of the pilus keeps its value, the pilus elongates towards the same direction in the $x - y-$plane as before, but with its z-component positioned exactly at the surface. If the length $l_k^{(\mathrm{fr})}$ of a free pilus (in this case the free length has the same value as the contour length) is smaller than 0 the pilus is removed. Furthermore a pilus is not able to switch back to the protrusion state after retraction started.

Attachment to a substrate or other pili

The binding to the substrate is controlled by a stochastic process characterized by the rate λ_sub. A pilus will always bind with its end point to a point $\mathbf{x}_k^{(\mathrm{e})} = \mathbf{x}_k^{(\mathrm{ps})}$ at the surface. The binding of two non-attached pili is described in a similar manner, however, in this case it is necessary to describe the binding of two pili in three-dimensional space. We account for a larger binding probability due to thermal fluctuations of a pilus by

using the beam-equation for a semi-flexible rod [2]. Solving the beam equation for pilus k gives a mean diameter d_{beam} of a cone as a function of the distance l from the start point $\mathbf{x}_k^{(\text{s})}$ of the form

$$d_{\text{beam}}(l) = \sqrt{\frac{l^3}{3l_{\text{p}}}}, \tag{S4}$$

with l_{p} denoting the persistence length. In our model two pili bind stochastically with the rate λ_{pil} if one free pilus, described as the cone (see equation (S4)), and a neighboring free pilus, described as a finite line, intersect. An intersection point $\mathbf{x}_k^{(\text{pp})}$ is picked randomly on this intersection interval. The intersection point is used to compute the lengths of the pili. The distance between the start point of a pilus $\mathbf{x}_k^{(\text{s})}$ and the intersection point $\mathbf{x}_k^{(\text{pp})}$ is given by $l_k^{(\text{int})}$. If this length is smaller than the free length of the pilus $l_k^{(\text{int})} \leq l_k^{(\text{fr})}$, binding will take place. Otherwise the binding will not take place because the pilus would need to be longer than its actual length. In case of binding the length of the tail $l_k^{(\text{tail})} = l_k^{(\text{fr})} - l_k^{(\text{int})}$ needs to be saved and will be added to the pilus after detachment. The new contour and free length $l_k^{(\text{con})}$ and $l_k^{(\text{fr})}$ of the pilus are defined to be $l_k^{(\text{int})}$, the distance from the start point to the attachment point. The binding of pili, similarly to the creation of new pili, is not checked for every time step, but only after $\Delta t_{\text{d}} = 0.02$ s.

Pilus forces

The pilus is modeled as a Hookean spring with a spring constant k_{pull} [3], which allows to compute the pulling force acting on the cell. The force of a pilus k attached to the substrate is given by

$$\mathbf{F}_{\mathbf{k}}^{(\mathbf{ps})} = \max\left[0,\ k_{\text{pull}} \cdot \left(l_k^{(\text{con})} - l_k^{(\text{fr})}\right) \cdot \frac{\mathbf{x}_k^{(\text{se})}}{\left|\mathbf{x}_k^{(\text{se})}\right|}\right] \tag{S5}$$

where $\mathbf{x}_k^{(\text{se})} = \mathbf{x}_k^{(\text{e})} - \mathbf{x}_k^{(\text{s})}$. Similarly the pulling force of a pilus k attached to another pilus j is given by

$$\mathbf{F}_{\mathbf{k}}^{(\mathbf{pp})} = \max\left[0,\ k_{\text{pull}} \cdot \left(l_k^{(\text{con})} - l_k^{(\text{fr})} - l_j^{(\text{fr})}\right) \cdot \frac{\mathbf{x}_k^{(\text{se})}}{\left|\mathbf{x}_k^{(\text{se})}\right|}\right]. \tag{S6}$$

During our simulations we set the end point of an attached pilus to the start point of the associated pilus in order to easily compute the direction vector $\mathbf{x}_k^{(\text{se})}$, thus the contour length of the single pilus $l_k^{(\text{con})}$ is now the contour length of the bundle.

Since the direction of pili forces between two bound pili only depend on $\mathbf{x}_k^{(\text{se})}$, it is sufficient to compute the distance of the start points of the two coupled pili and compare it to the sum of their free lengths. While the contour length solely depends on the motion of the cells and the position of the pili start points, the free length of pilus k is changed due to its retraction. The retraction velocity depends on the pulling force:

$$v_k^{(\text{ret})}(F) = \max\left[0,\ v_{\text{ret}} \cdot \left(1 - \frac{F}{F_{\text{stall}}}\right)\right]. \tag{S7}$$

Here F is either the absolute value of a the pili-pili forces $F = \left|\mathbf{F}_{\mathbf{k}}^{(\mathbf{pp})}\right|$, or the absolute value of a force resulting from an attachment to the substrate $F = \left|\mathbf{F}_{\mathbf{k}}^{(\mathbf{ps})}\right|$. F_{stall} is the stalling force and determines the characteristic pulling force of a pilus, because the retraction of pili is stalled for larger forces $F > F_{\mathrm{stall}}$ [4]. The characteristic pulling force enables us to neglect the length-dependence of the spring constant of the pili. By using a very large spring constant k_{pull} (relative to the F_{stall}), this force governs the dynamics of the system and not the spring constant itself. While pili that fulfill the condition $l_k^{(\mathrm{con})} > F_{\mathrm{stall}}/k_{\mathrm{pull}}$ are able to generate forces in the order of the characteristic force F_{stall}, the pulling force of shorter pili is no longer able to exceed. While a high spring constant guarantees that the resulting pili need to be extremely short, we set the pulling force of a short pilus equal to the stalling force. This is motivated by the fact that the time to reach the stalling force for a pilus is proportional to its length. The force-dependent binding of pili, similarly to the creation of new pili or their binding, is not checked for every time step, but only after $\Delta t_{\mathrm{d}} = 0.02$ s.

Cell forces and motility

An intersection of two cocci of two different cells causes a repulsive force. For the sake of simplicity we call the first coccus i (position vector $\mathbf{r}_i^{(\mathrm{a})}$) and the second coccus j (position vector $\mathbf{r}_j^{(\mathrm{b})}$). The force is

$$\mathbf{F}_{ij}^{(\mathrm{cc})} = -k_{\mathrm{cc}} \left(2R - |\mathbf{r}_{ij}|\right) \frac{\mathbf{r}_{ij}}{|\mathbf{r}_{ij}|}, \tag{S8}$$

with $\mathbf{r}_{ij} = \mathbf{r}_i^{(\mathrm{a})} - \mathbf{r}_j^{(\mathrm{b})}$ and the excluded volume spring constant k_{cc}. A similar equation describes the contribution of the substrate which is located at $z = 0$. If a coccus i (with position vector $\mathbf{r}_i^{(\mathrm{j})}$, $j = a, b$) intersects with the substrate and overlaps by a distance Δd_{ov}, a repulsive force is acting on the coccus:

$$\mathbf{F}_i^{(\mathrm{cs})} = k_{\mathrm{cs}} \Delta d_{\mathrm{ov}} \mathbf{e}_{\mathrm{z}}. \tag{S9}$$

Here, k_{cs} denotes the excluded volume spring constant and $\mathbf{e}_{\mathrm{z}}$ is the unit vector pointing perpendicular to the substrate. The force does not act on the surface point, but at the point $\mathbf{r}_i^{(\mathrm{cs})} = \mathbf{r}_i^{(\mathrm{j})} - z_{\mathrm{k}} \cdot \mathbf{e}_{\mathbf{z}}$ with the z-component $z_{\mathrm{k}} = \mathbf{r}_i^{(\mathrm{j})} \cdot \mathbf{e}_{\mathbf{z}}$ of the coccus. This is realized by a very high spring constant of the surface and a small overlap. In this case, the torque will only be weakly affected.

Simulation details and parameters

Our simulations were performed on the local computing cluster consisting of x86-64 GNU/Linux systems of the MPI-PKS. All machines possess Intel Xeon processors with a clock rate of 2.2 to 3.0 GHz and have between 2 to 4 CPUs. The code was written in C++ and parallelized on CPU by using the library OpenMP. We used the GCC-compiler (version 4.8.1) and were running the simulations on 8 cores in parallel.

S3. Fitting Results

S3.1. Velocity autocorrelation of single cells on a surface

We fitted a double-exponential function of the form

$$C(\tau) = \langle \mathbf{v}(t+\tau) \cdot \mathbf{v}(t) \rangle_t = v_1^2 \cdot \exp\left(-\frac{\tau}{\tau_1}\right) + v_2^2 \cdot \exp\left(-\frac{\tau}{\tau_2}\right) \tag{S10}$$

to the velocity autocorrelation data. The results of the fitting for strong and weak pili-substrate interactions are given in table S1.

S3.2. Profile of diffusion coefficient of cells inside of colony

We fitted an exponential function of the form

$$D(d_{\mathrm{com}}) = D_0 + D_r \exp\left(\frac{d_{\mathrm{com}}}{d_{\mathrm{grad}}}\right) \tag{S11}$$

to the diffusion coefficient D of cells as a function of their distance from the center of mass d_{com} of the colony. The results of the fitting for strong and weak pili-substrate interactions are given in table S2.

S3.3. Profile of cell densities of a spherical colony

We fitted a function of the form

$$\rho(d_{\mathrm{com}}) = \frac{\rho_0}{2} \cdot \left[1 - \tanh\left(\frac{d_{\mathrm{com}} - R_{\mathrm{col}}}{\omega}\right)\right] \tag{S12}$$

to the density of cells ρ as a function of their distance from the center of mass d_{com} of the colony. The results of the fitting for strong and weak pili-pili interactions are given in table S3.

Table S1. Parameters for the double exponential fit of velocity autocorrelation

Set	$v_1[\mu\mathrm{m/s}]$		$v_2[\mu\mathrm{m/s}]$		$\tau_1[\mathrm{s}]$		$\tau_2[\mathrm{s}]$	
Strong	0.26	± 0.01	0.06	± 0.01	0.08	± 0.01	5.05	± 0.40
Weak	1.17	± 0.03	0.13	± 0.03	0.15	± 0.04	1.56	± 0.08

Table S2. Parameters for the fit of the diffusion coefficient of a cell within a colony as a function of the distance from the center of the colony

Set	$D_0[\mu\mathrm{m}^2/\mathrm{s}]$		$D_\mathrm{r}[\mu\mathrm{m}^2/\mathrm{s}]$		d_{grad}	
Strong	$6.6 \cdot 10^{-5}$	$\pm 0.9 \cdot 10^{-5}$	$1.9 \cdot 10^{-7}$	$\pm 2.0 \cdot 10^{-7}$	0.98	± 0.17
Weak	$2.0 \cdot 10^{-3}$	$\pm 0.3 \cdot 10^{-3}$	$5.7 \cdot 10^{-6}$	$\pm 3.2 \cdot 10^{-6}$	1.06	± 0.31

Table S3. Parameters for the tanh fit of the cell density inside of a colony

Set	$\rho_0[1/\mu m^3]$	$R_{col}[\mu m]$	$\omega[\mu m]$
Strong	0.20 ± 0.01	7.97 ± 0.02	0.18 ± 0.03
Weak	0.17 ± 0.02	8.19 ± 0.02	0.40 ± 0.02

S4. Simulation Details

If not mentioned otherwise we used the parameter set, given in table S4.

S4.1. Single Cells

We place an individual cell on top of substrate with a distance similar to the cell radius and random cell orientation. For the first 2 seconds of the simulations the cells only interacted via the repulsive excluded volume interactions to remove overlaps of the randomly distributed cells. After this time we turned on the production and dynamics of pili. To reduce the impact of the initial condition on our results we simulated a time span of 30 minutes and analyzed the last 20 minutes. For every parameter set we analyzed the trajectories of 250 cells and sampled over the parameter set given in table S5.

S4.2. Single Colony on Substrate

We initialized a colony by randomly distributing N cells (with random orientations) in a sphere with radius $0.85 \cdot N^{1/3}$ μm at a position far enough from the substrate so that every cell will have a distance of at least 2.5 μm from the substrate, which is larger

Table S4. Parameters for the simulations

Parameter	Value	Reference
Time step Δt	$5 \cdot 10^{-6}$ s	
Time step of pilus state change Δt_{d}	$2 \cdot 10^{-2}$ s	
Cocci Radius R	0.5 μm	[5]
Cocci Distance d	0.6 μm	[5]
Cell-Cell excl. vol. const. k_{cc}	$2 \cdot 10^{4}$ pN/μm	
Cell-Sub excl. vol. const. k_{cs}	$4 \cdot 10^{4}$ pN/μm	
Translational mobility μ_{trans}	1 μm/(s $\cdot$ pN)	
Rotational mobility μ_{rot}	2 $(\mathrm{s} \cdot \mathrm{pN})^{-1}$	
Pili persistence length l_{p}	5 μm	[6]
Pili production rate λ_{p}	15 Hz	
Maximal pili number N_{pili}	15	[7]
Pili protrusion velocity v_{pro}	2 μm/s	[5, 8]
Pili retraction velocity v_{ret}	2 μm/s	[5, 8]
Pili retraction rate λ_{ret}	1.33 Hz	[5]
Pili spring constant k_{pull}	2000 pN/μm	[3]

Table S5. Parameter Sampling for single cells on substrate

Parameter	Value
Pili substrate attachment rate λ_{sub} [Hz]	0.25, 0.5, 1, 2
Pili substrate detachment force $F_{\mathrm{d,sub}}$ [pN]	60, 120, 180, 300, 360
Pili substrate detachment time $t_{\mathrm{d,sub}}$ [s]	5, 10, 30, 60

Table S6. Parameters for colonies on a substrate

Parameter	Value
Cell number N	1, 5, 10, 25, 50, 100, 300, 1600
Pili pili attachment rate λ_{pil} [Hz]	0.5
Pili substrate attachment rate λ_{sub} [Hz]	0.5
Pili substrate detachment force $F_{\text{d,sub}}$ [pN]	180, 300
Pili substrate detachment time $t_{\text{d,sub}}$ [s]	10, 30
Pili pili detachment force $t_{\text{d,pil}}$ [pN]	360
Pili pili detachment time $t_{\text{d,pil}}$ [s]	50

Table S7. Parameters for free colonies

Parameter	Value
Cell number N	1700
Pili pili attachment rate λ_{pil} [Hz]	0.25, 0.5, 2
Pili pili detachment force $t_{\text{d,pil}}$ [pN]	120, 180, 240, 300, 360
Pili pili detachment time $t_{\text{d,pil}}$ [s]	5, 20, 30, 40, 50, 60, 70

than the characteristic pili length but not too large such that the pili would not be able to attach to the substrate. We allowed initial overlap of the cells and only activated excluded volume forces for the first 2 seconds of the simulation. Afterwards we activated the pili and simulated for 20 minutes, but only analyzed the last 10 minutes. For every parameter set (given in table S6) we analyzed the trajectories of 10 colonies.

S4.3. Free Single Colony

We initialized a colony by randomly distributing 1700 cells (with random orientations) in a sphere with radius of approximately 10μm. For the first 2 seconds we only activated the excluded volume forces in order to remove overlaps of the cells. After this time we switched on the production and the dynamics as described in the main text (see section 2). We simulated *in silico* colonies for 30 minutes and analyzed the properties of the colonies for the last 20 minutes. For every parameter set (see table S7) we analyzed a single colony. For those sets that are presented in the paper we analyzed 10 different realizations.

S4.4. Colony Coalescence

We initialized two colonies by randomly distributing N cells (with random orientations) per colonies in two sphere with radius $0.85 \cdot N^{1/3}$ μm and distances slightly larger than the sum of the radii of the colonies. For the first 2 seconds we only activated the excluded volume forces in order to remove overlaps of the cells. For the following 100 s we only allowed pili interactions between the cells of the individual colonies in order to create stable colonies. After this time we activated the interactions of pili of both colonies so

Table S8. Parameters for coalescence

Parameter	Value
Cell number per colony N	1000
Pili pili attachment rate λ_{pil} [Hz]	0.25, 0.5, 2
Pili pili detachment force $t_{\mathrm{d,pil}}$ [pN]	120, 180, 240, 300, 360
Pili pili detachment time $t_{\mathrm{d,pil}}$ [s]	5, 20, 30, 40, 50, 60, 70

Table S9. Parameters for Assembly

Parameter	Value
Cell number N	1200
System size $L \times L$ [μm $\times$ μm]	88×88
Pili pili attachment rate λ_{pil}	0.25, 0.5, 1, 2
Pili pili detachment force $t_{\mathrm{d,pil}}$ [pN]	360
Pili pili detachment time $t_{\mathrm{d,pil}}$ [s]	50
Pili substrate detachment force $F_{\mathrm{d,sub}}$ [pN]	180, 300, 360
Pili substrate detachment time $t_{\mathrm{d,sub}}$ [s]	5, 10, 30, 60
Ratio of $\Delta pilT$ mutants γ_{Δ}	0, 0.1, 0.5

that the coalescence could start. We simulated the *in silico* colonies for 30 minutes and analyzed the colonies starting from 60 seconds. For every parameter set we analyzed a single coalescence event (see table S8). For those sets that are presented in the paper we analyzed 14 different realizations (i.e. for the size-dependence of the coalescence).

S4.5. Assembly on a substrate

We initialized 1200 randomly oriented cells on the substrate with a relative distance similar to the cell radius. A fraction γ_{Δ} of these cells have pili that are not able to retract. For the first two seconds cells only interact via excluded volume forces until all overlaps of the cells with each other and the substrate have vanished. After this time we switched on all pili-mediated interactions and all related interactions of the cells. We simulated a total duration of 60 minutes. The system is characterized by periodic boundary conditions along both axes parallel to the substrate plane with a size 88×88 μm. For every parameter set (see table S9) we computed a single assembly realization.

S5. Data Analysis

We used Matlab R2015b and its internal functions to analyze the data.

S5.1. Single Cells

In order to compute the velocity at time t of a cell moving along the trajectory $\mathbf{r}(t)$ we computed the displacement $\Delta\mathbf{r}(t) = \mathbf{r}(t+\tau) - \mathbf{r}(t)$ and set the velocity $\mathbf{v}(t) = \Delta\mathbf{r}(t)/\tau$ with $\tau = 0.1$ s.

S5.2. Single Colony on Substrate

The radii of microcolonies moving over a substrate were computed by projecting the shape of the individual cells onto the plane of the substrate. This two-dimensional projection exhibits to a good approximation a circular shape from which we extracted the radius $R(t)$ of the colony. By averaging over both the time and different realisations of colonies we calculated the mean colony radius as a function of the number of individual cells within a colony. Additionally, we checked that the mean radius was not affected by the sampling time interval of the time average, for example due to the loss of individual cells from the colony. The diffusion coefficient D of the colonies were determined from the mean-squared-displacement

$$\langle (\mathbf{r}_{\mathrm{col}}(t+\tau) - \mathbf{r}_{\mathrm{col}}(t))^2 \rangle_t = 4D\tau, \tag{S13}$$

where $\mathbf{r}_{\mathrm{col}}(t)$ denotes the center of the projected two-dimensional colony $\mathbf{r}_{\mathrm{col}}(t)$. The projection onto the plane of the substrate as discussed above allows us to define the radius and the position of a colony similar to experiments, in which one would detect the edges of a colony and analyze the corresponding binary image [9]. Similar to the computation of the projected colony shape on the substrate we defined the shape of the side-view of the colony by projecting the colony onto a plane perpendicular to the surface and computing its average shape by thresholding the sum of the individual areas of the different colonies.

S5.3. Free Single Colony

In order to compute the diffusion coefficients of cells within a microcolony as a function of their distance from the COM of the microcolony we introduced multiple shells defined by a minimal distance d_{min} and d_{max} from the the center of the microcolony. Afterwards, we picked those parts of the trajectory $\mathbf{r}_i(t)$ for which cell i solely moved inside of a single shell for at least 10s. From these trajectories we computed the time-averaged mean squared displacement (which was additionally averaged over all cells i) and estimated the diffusion coefficients D from the relation

$$\langle (\mathbf{r}_i(t+\tau) - \mathbf{r}_i(t))^2 \rangle_{i,t} = 6D\tau. \tag{S14}$$

S5.4. Colony Coalescence

In order to compute the height of the bridge forming between two colonies and the properties of an ellipse fitted to their shape, we projected the diplococcus shape of the individual cells onto a plane parallel to the axis between the centers of the two colonies. From the projection of the individual cells we can compute the envelope of the two colonies.

In order to compute the bridge height we defined a line of length 1μm centered around the COM of the projected envelope and parallel to the axis connecting the centers of the two colonies. We moved this line perpendicular to the axis between the colony centers and defined the height of the bridge as the range for which the whole line is inside of the colony envelope. The length of the line was chosen to be small enough so that it was not affected by the circular shape of the projection and large enough so that the effects of the fluctuations of single cells close to the surface of the colonies were reduced. For the late coalescence the results were compared to the short axis of the ellipse. By computing the central moments of the projected two-dimensional area of the microcolonies we were able to compute the properties of the ellipse as explained in [10]. This method allowed us to measure the bridge height and the ellipse properties in a similar manner to the analysis of experimental data.

S5.5. Assembly on a substrate

To define which cells are part of the surface and which cells are part of the bulk of a microcolony moving over a surface, we computed the alpha shape of the collection of cocci points of all cells [11]. The underlying idea of this triangulation technique is the motion of a sensor sphere over the surface of the colonies and the individual cells. Such a sphere is not allowed to intersect the positions of the cocci. If any coccus of a cell is touched by such a sphere (thus laying on the surface of the sphere), the corresponding cell is defined to be a surface cell. The only free parameter is the radius $R_\alpha = 1$ μm which is defined to be in the order of the cell sizes. More details of this method are given in reference [11].

S6. References

[1] Safran S 2003 *Statistical Thermodynamics of Surfaces, Interfaces, and Membranes* Frontiers in physics (Westview Press)

[2] Howard J 2001 *Mechanics of Motor Proteins and the Cytoskeleton* (Massachusetts, USA: Sinauer Associates, Inc.)

[3] Biais N, Higashi D, Brujic J, So M and Sheetz M 2010 *Proc. Nat. Acad. Sci. USA* **107**(25) 11358–11363

[4] Maier B 2013 *Soft Matter* **9**(24) 5667–5671

[5] Zaburdaev V, Biais N, Schmiedeberg M, Eriksson J, Jonsson A B, Sheetz M and Weitz D 2014 *Biophys. Jour.* **107**(7) 1523–1531

[6] Skerker J and Berg H 2001 *Proc. Nat. Acad. Sci. USA* **98**(12) 6901–6904

[7] Chang Y W, Rettberg L A, Treuner-Lange A, Iwasa J, Søgaard-Andersen L and Jensen G J 2016 *Science* **351**(6278) aad2001

[8] Marathe R, Meel C, Schmidt N C, Dewenter L, Kurre R, Greune L, Schmidt M A, Müller M J, Lipowsky R, Maier B and Klumpp S 2014 *Nature Comm.* **5**

[9] Taktikos J, Lin Y T, Stark H, Biais N and Zaburdaev V 2015 *PloS One* **10**(9) e0137661

[10] Burger W and Burge M J 2009 *Principles of Digital Image Processing - Core Algorithms* (Springer)

[11] Edelsbrunner H, Kirkpatrick D and Seidel R 1983 *IEEE T. Inform. Theory* **29**(4) 551–559
